# Analysis of N-linked Glycan Alterations in Tissue and Serum Reveals Promising Biomarkers for Intrahepatic Cholangiocarcinoma

**DOI:** 10.1158/2767-9764.CRC-22-0422

**Published:** 2023-03-06

**Authors:** Shaaron Ochoa-Rios, Calvin R.K. Blaschke, Mengjun Wang, Kendell D. Peterson, Andrew DelaCourt, Stéphane Elie Grauzam, David Lewin, Peggi Angel, Lewis R. Roberts, Richard Drake, Anand S. Mehta

**Affiliations:** 1 Department of Cell and Molecular Pharmacology, Medical University of South Carolina, Charleston, South Carolina.; 2 Department of Pediatrics, Medical University of South Carolina, Charleston, South Carolina.; 3 Department of Pathology and Laboratory Medicine, Medical University of South Carolina, Charleston, South Carolina.; 4 Division of Gastroenterology and Hepatology, Mayo Clinic, Rochester, Minnesota.

## Abstract

There is an urgent need for the identification of reliable prognostic biomarkers for patients with intrahepatic cholangiocarcinoma (iCCA) and alterations in N-glycosylation have demonstrated an immense potential to be used as diagnostic strategies for many cancers, including hepatocellular carcinoma (HCC). N-glycosylation is one of the most common post-translational modifications known to be altered based on the status of the cell. N-glycan structures on glycoproteins can be modified based on the addition or removal of specific N-glycan residues, some of which have been linked to liver diseases. However, little is known concerning the N-glycan alterations that are associated with iCCA. We characterized the N-glycan modifications quantitatively and qualitatively in three cohorts, consisting of two tissue cohorts: a discovery cohort (*n* = 104 cases) and a validation cohort (*n* = 75), and one independent serum cohort consisting of patients with iCCA, HCC, or benign chronic liver disease (*n* = 67). N-glycan analysis *in situ* was correlated to tumor regions annotated on histopathology and revealed that bisected fucosylated N-glycan structures were specific to iCCA tumor regions. These same N-glycan modifications were significantly upregulated in iCCA tissue and serum relative to HCC and bile duct disease, including primary sclerosing cholangitis (PSC) (*P* < 0.0001). N-glycan modifications identified in iCCA tissue and serum were used to generate an algorithm that could be used as a biomarker of iCCA. We demonstrate that this biomarker algorithm quadrupled the sensitivity (at 90% specificity) of iCCA detection as compared with carbohydrate antigen 19-9, the current “gold standard” biomarker of CCA.

**Significance:**

This work elucidates the N-glycan alterations that occur directly in iCCA tissue and utilizes this information to discover serum biomarkers that can be used for the noninvasive detection of iCCA.

## Introduction

Cholangiocarcinoma (CCA) is an epithelial cancer arising in the biliary mucosa lining the ducts that carry bile from the liver to the small intestine (1). CCA is the second most common type of liver cancer after hepatocellular carcinoma (HCC) and is a highly lethal cancer (2). CCA is classified on the basis of the anatomic location into intrahepatic CCA (iCCA), perihilar CCA (pCCA), or distal CCA (dCCA) subtypes (3). CCAs frequently consist of small nests of epithelial cancer cells surrounded by dense stromal regions of cancer-associated fibroblasts, immune cell populations, and extracellular matrix. In addition, glandular formations are also a histologic characteristic of CCA (4). CCA risk factors can be subtype specific: diabetes, obesity, smoking, chronic viral hepatitis, cirrhosis, chronic pancreatitis, and chemical exposure have been linked to iCCA compared with other subtypes (2, 3, 5). Other risk factors have been associated with geographic regions, where primary sclerosing cholangitis (PSC) is the most common risk factor in Western countries and liver fluke infection in Southeast Asia (5). Despite advances made so far in understanding the risk factors and biological mechanisms of the disease, a definitive diagnosis of CCA at an early stage continues to be challenging. For CCA diagnosis, a combination of diagnostic methods is necessary and a biopsy should be performed when feasible and taken into consideration for the final diagnosis (3). Conventional diagnostic techniques do not account for the heterogeneity of the tumor location, size, and pathologic and cellular characteristics (1, 2). Consequently, there is an urgent need for improved biomarkers and treatments for CCA.

Glycosylation, one of the most common post-translational modifications, regulates biological functions including cell-cell communication, protein folding, and receptor signaling (1, 6). Dysregulation in glycosylation has been reported in many cancers, including CCA (1, 7–9). N-linked glycosylation is the addition of glycan structures to the glycoprotein at the asparagine residue. This process occurs through a well-established biosynthetic processing pathway in the endoplasmic reticulum and Golgi apparatus. The composition of these N-glycan structures is influenced by the abundance of glycosyltransferases and glycosidases, which add and remove monosaccharides, respectively, as well as the availability of nucleotide-monosaccharide donors (6, 10). N-glycan alterations like fucosylation (addition of a fucose residue), sialyation (addition of a sialic acid residue), and complex branching (addition of a GlcNAc residue) have well-established associations with many different cancers, including HCC (11–14). Alterations in fucosylation can be classified as (i) core fucosylation or (ii) outer-arm fucosylation. Fucosyltransferases (FUT 1–11) catalyzes the addition of the respective fucose residue at a specific linkage of the N-glycan structure. FUT8 is the only enzyme capable of adding a fucose residue at the α1–6 linkage of the N-glycan structure (known as core fucosylation). While the remaining FUTs can add fucose residues at different linkages on the antenna of the N-glycan structure (known as outer-arm fucosylation). Because of the many biological roles, N-glycosylation is responsible for, N-glycan structures can also be altered in healthy tissue. We have previously reported N-glycan alterations like high mannose, and biantennary N-glycans with limited fucosylation and branching in healthy liver tissue (15). N-glycosylated antigens have been widely used as biomarkers for different types of cancers: alpha-fetoprotein (AFP) for HCC, PSA for prostate cancer, and carbohydrate antigen 19-9 (CA19-9) for CCA. However, the low specificity of CA19-9 reduces its clinical utility, and a higher-performing biomarker for CCA is urgently needed. Previous research has identified alterations in the abundance and/or N-glycosylation of certain serum or plasma glycoproteins that are correlated to liver damage caused by CCA and CCA tumor progression (1, 8, 9). We, and others, have previously correlated changes in glycosylation in the tissue and serum of individuals with the development of HCC (15–22). However, it is unclear if a similar change would be observed in the other major type of liver cancer, CCA.

To address this gap, we utilized matrix-assisted laser desorption ionization (MALDI) imaging mass spectrometry (IMS) for the characterization of the N-glycan–related molecular changes occurring in tissue and serum. A total of three cohorts were analyzed, consisting of two tissue cohorts: a discovery cohort (*n* = 104 cases) and a validation cohort (*n* = 75), and one independent serum cohort consisting of patients with iCCA, HCC, or benign chronic liver disease (*n* = 67). Finally, we use the identified N-glycomic profiles for the development of a potential biomarker that could distinguish iCCA from other types of liver damage (23, 24).

## Materials and Methods

### Tissues and Tissue Microarrays

Initial analysis was performed using normal liver tissue (HuFPT074), HCC tissue (HuCAT081), and iCCA tissue (HuCAT086; Biomax, Inc.). Hematoxylin and eosin (H&E) stains of each tissue were annotated by a pathologist with the tumor, adjacent to the tumor, and fibrotic regions. Subsequently, analysis was performed using a tissue microarray (TMA; #LV2081, Biomax, Inc) that contained 208 cores with 103 cases (duplicated cores per case): consisting of fifty HCC, twenty iCCA, one clear-cell carcinoma cyst, five metastatic HCC (spleen, chest wall, cerebrum, costal bone, and lymph node), two hepatic cyst, eight tissues with cirrhosis and dysplastic nodules, ten hepatitis-infected tissues, two adjacent normal tissues, and six independent normal tissues. The cores were 1.0 mm in diameter and validated by pathology. The validation tissue cohort TMA (DLV753, US Biolab) contained 75 cases with 75 cores: forty-five cases of HCC, twenty-three cases of iCCA, two cases of mixed carcinoma, and five cases of normal liver tissue. Cores were 1.5 mm in diameter and validated by pathology. All tissue samples were formalin-fixed paraffin-embedded (FFPE) cut into a 5-μm-thick section and unstained before analysis. H&E stainings of all tissues and TMAs annotated by a pathologist for small duct and large duct classifications for each core are provided in Supplementary Fig. S1A–C. Clinical information for TMAs is listed in Supplementary Fig. S2A and S2B.

### Serum Samples

Samples were from 30 patients with iCCA, 17 samples with PSC, and 20 patients with other liver diseases but not iCCA or PSC seen at Mayo Clinic, Rochester, MN between January 2000 and May 2010. Peripheral blood was collected from each participant at the time of the office visit before treatment. Sera were stored at −80°C. The following data elements were abstracted from the medical record: demographics (age, gender, ethnicity, race, weight, height), medical history, etiology of liver disease, laboratory data including CA19-9 and AFP, and imaging results (ultrasound, CT, or MRI). Histopathology results and radiologic findings from the medical records of all patients were reviewed to ascertain the diagnosis of iCCA and identify tumor location. The diagnosis of iCCA in all patients was confirmed by histopathology. The anatomic location of CCAs was categorized as “intrahepatic” if the mass lesion arose within the hepatic parenchyma and did not extend to or involve the secondary branches of the biliary trees as demonstrated either by CT imaging, MRI, or endoscopic retrograde cholangiopancreatography findings. The etiology of liver disease was based on the laboratory, imaging, and histopathology results and the judgment of the treating physician. For patients with viral hepatitis, anti-HCV antibody, serum HCV RNA, HBV surface antigen, HBV e-antigen, and HBV DNA levels were recorded. Clinical information of the serum cohort is listed in Supplementary Fig. S2C.

### Enzymes and Reagents

Trifluoroacetic acid, Harris-modified hematoxylin, and α-cyano-4-hydroxycinnamic acid (CHCA) were obtained from Sigma-Aldrich. High-performance liquid chromatography (HPLC) grade methanol, ethanol, aceto-nitrile, xylene, hydrogen peroxide, and water were obtained from Thermo Fisher Scientific. Recombinant peptide N-glycosidase F (PNGase F) PRIME and endoglycosidase F3 Prime (Endo F3) were obtained from N-Zyme Scientifics.

### Tissue Preparation for MALDI-IMS

Unstained FFPE tissue slides were processed using standardized N-glycan imaging workflows of MALDI-IMS as described previously (15, 25) Briefly, tissues were heated at 60°C for 1 hour and cooled to room temperature before deparaffinization. The slides were washed with xylene to remove the paraffin and then rehydrated using a series of water and ethanol washes. Antigen retrieval was performed using citraconic anhydride (Thermo Fisher Scientific) as the buffer and placed in a decloaker for 30 minutes. The buffer was then cooled to room temperature and buffer exchange was performed to replace the slides in 100% water. To release the N-glycans, an M5 TM-Sprayer Tissue MALDI Sample Preparation System (HTX Technologies, LLC) was used to spray 0.5 mL of 0.1 μg/μL aqueous solution PNGase F PRIME (N-zyme Scientifics) as described previously (15, 26). To elucidate the core fucosylated N-glycan profile, we treated the validation TMA with the enzyme Endo F3 Prime, which preferentially cleaves core-fucosylated N-glycans between the two core N-acetylglucosamine residues and results in a mass shift of 349.137 m/z for core fucosylated N-glycans compared with N-glycans released only by PNGaseF (27). Following the spray of the respective enzyme, the slides were placed in a humidified chamber and incubated at 37°C for 2 hours. Slides were then desiccated and dried before matrix application. The matrix was assembled using CHCA (0.042 g CHCA in 6 mL 50% acetonitrile/49.9% water/0.1% trifluoroacetic acid) and sprayed using the same M5 TM-Sprayer.

### Serum Preparation for MALDI-IMS

Serum samples were processed for N-glycan analysis as described previously (28). Briefly, 1 μL of serum was diluted in 2 μL of sodium bicarbonate (100 mM, pH 8.0). After mixing, 1 μL was spotted on a hydrogel-coated slide. Serum samples were spotted in triplicate and a blank well was always included in the same slide as the serum samples. Spots were left to immobilize onto the slide at room temperature for 1 hour and washed a total of three times with Carnoy solution and one time with double distilled water. PNGase F PRIME and matrix were sprayed for tissue samples described above.

### N-glycan Imaging Using MALDI-IMS

All tissues and serum samples were imaged using a timsTOF Flex MALDI-QTOF mass spectrometer (Bruker Daltonics); (m/z 500–4000) operating in a positive mode. Focus Pre TOF parameters were set as followed: transfer time 120.0 μs and pre-pulse storage 25.0 μs. For whole tissues (normal, HCC, and iCCA) and TMAs images were collected at 100 μm raster. For serum samples, images were collected at 150 μm raster. All images were collected at 200 laser shots per pixel.

### Data Processing

Data analysis was done in SCiLs lab 2021 imaging software (Bruker) for analysis of the mass range m/z 500 to 4000. SCiLs-generated N-glycan spectras were normalized to the total ion count. N-glycan structure annotations for each m/z (mass to charge ratio) value were made based on an in-house database of known N-glycan generated using GlycoWorkBench and GlycoMod for annotation or to MS-MS data previously done by our group (29, 30). For tissue, the maximum mean value of each m/z was extracted and used to calculate the relative intensities for each of the N-glycan peaks identified. For serum analysis, the following steps were applied to the extracted maximum mean value data: a blank well was included in the experimental plan and was subtracted from each N-glycan. Only N-glycans present in at least 80% of the samples were used for analysis. The standard sample was used to create normalization factors for each N-glycan on each slide, where the intensity of the individual slide's standard was divided by the average intensity of the standards across all slides for each N-glycan. Each slide's N-glycans were then multiplied by the corresponding normalization factor and the glycan intensities were converted to the relative intensity. A list of all N-glycans identified in tissue and serum can be found in Supplementary Table S1.

### Statistical Analysis

N-glycan data was explored with a boxplot and scatter plot (outliers were included). Descriptive statistics were presented as mean values ± SD unless otherwise stated. Statistical inference between two groups was applied with a *t* test or Mann–Whitney test based on the distribution of the data, normality of data was checked by the Shapiro–Wilk test. To explore the statistical inference of associations between outcomes with glycans, we applied logistic regression for a binary outcome, ordinal logistic regression, tree analyses, and random forest algorithm for multiple ordinal outcomes. Logistic regression was also used to derive the rate of change and corresponding *P* value of each glycan change from those without iCCA to those with iCCA in both TMA and serum. ROC curves were built to assess the discriminating ability of individual glycans or panels of glycans. The area under the ROC curve (AUC) was also chosen as a criterion in each step of feature selection to remove noninformative glycans or redundant glycans during optimization.

To alleviate the bias of feature selection, we applied various methods such as stepwise logistic regression, Lasso algorithm, correlation filter, random forest algorithm for feature selection to optimize the combination of glycans, and cross-validations [leave-one-out cross-validation (LOOCV) and 3-fold CV] were further applied during feature selection to avoid bias (31). On the basis of the robustness, interpretability, and suitability for the glycan panel structure of interest, we chose a logistic regression model to build a predictive algorithm with selected features. The relative importance of the chosen glycans in the algorithm was derived. The performance of the predictive algorithm was explored by apparent validation (classification) and LOOCV. AUC was calculated, its SE was derived using a bootstrap method with 2,000 iterations, and the 95% confidence interval of AUC and corresponding *P* value were derived from the SE. Statistical comparison between two AUCs was performed using Delong test.

Cluster analysis was also applied to explore the similarity of the data structure of the glycans of interest based on statistical distances. Tanglegram (Cophylo plot) of serum and TMA were plotted to explore the congruence of the two dendrograms (32, 33). Principal component analysis (PCA) was used for further exploring glycan data structure based on its variation-covariance (information of each glycan), biplots of selected glycans of serum and TMA are provided for visual inspection in relationship of principal components.

Statistical analysis was performed by Graph Pad Prism 9.0 software package (Graph Pad, Inc) and R (version 4.1, https://www.r_proje.ct.org)

### Study Approval

Human serum samples were obtained from the Mayo Clinic Hepatobiliary Neoplasia Registry and Biorepository under an Institutional Review Board (IRB)-approved protocol. This study was approved by Mayo Clinic IRB and met all guidelines set forth by the 1975 Declaration of Helsinki for good clinical practice. All participants provided written informed consent for this study.

### Data Availability Statement

Datasets analyzed in this article are available upon request from the corresponding author.

### Supplementary Information

Additional supporting information is available in the online version of the article.

## Results

### N-glycan Alterations Correlate to Histopathologic Changes

To elucidate the *in situ* N-glycan changes that occur in iCCA tissue, we utilized MALDI-IMS methodology. Relative intensities across each tissue are presented via a heatmap of individual m/z (mass to charge ratio) values, where blue is low abundance and red is high abundance. Here, m/z values are representative of specific N-glycan structures.

**FIGURE 1:**
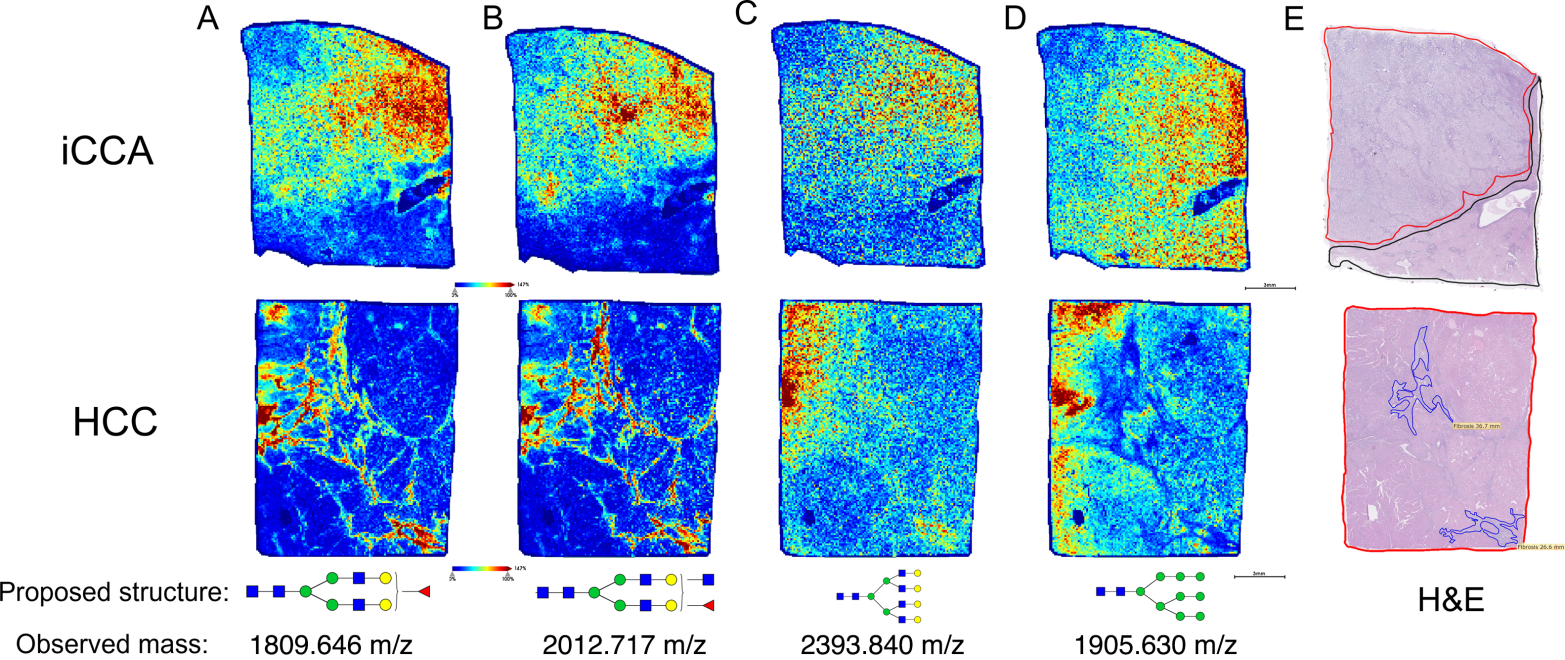
Bisected and biantennary fucosylated structures are highly expressed in iCCA tumor. Representative images of the relative intensity of biantennary fucosylated N-glycan (1809.646 m/z); (**A**) bisected fucosylated N-glycan (2012.717 m/z); (**B**) tetraantennary branched N-glycan (2393.840 m/z); (**C**) high mannose N-glycan (1905.630 m/z); (**D**) proposed N-glycan structures at the bottom correspond to the respective m/z value (observed mass). (**E**) H&E staining. Tumor regions are outlined in red, normal areas are outlined in black, and fibrotic regions are outlined in blue. Intrahepatic Cholangiocarcinoma (iCCA), and Hepatocellular Carcinoma (HCC). For N-glycans, red triangle, fucose; blue square, N-acetylglucosamine; green circles, mannose; yellow circles, galactose.

In Fig. 1, specific N-glycans that are altered with the development of iCCA, and HCC are shown based on the histopathologic changes annotated by a pathologist. N-glycans at m/z 1809.646 and 2012.717 (biantennary and bisected fucosylated N-glycans, respectively) were predominantly expressed in iCCA tissue, specifically in the tumor region while in the HCC tissue, these were present in only the fibrotic areas within the tumor region (Fig. 1A, B, and E). However, an N-glycan at m/z 2393.840 (complex highly branched N-glycan) was highly expressed in HCC tissue compared with iCCA and was also expressed in the tumor region of iCCA tissue but at a lower intensity (Fig. 1C–E). Finally, an N-glycan at m/z 1905.630 (high mannose N-glycan) did not show a definitive localization to the histology of iCCA or HCC tissues (Fig. 1D and E). Overall, we demonstrate by MALDI-IMS that the origin of specific N-glycan modifications can change based on the type of liver cancer or the histopathologic changes in each tissue.

### Bisected Fucosylated N-glycan Alterations are Specific to Patients with iCCA in Tissue

**FIGURE 2:**
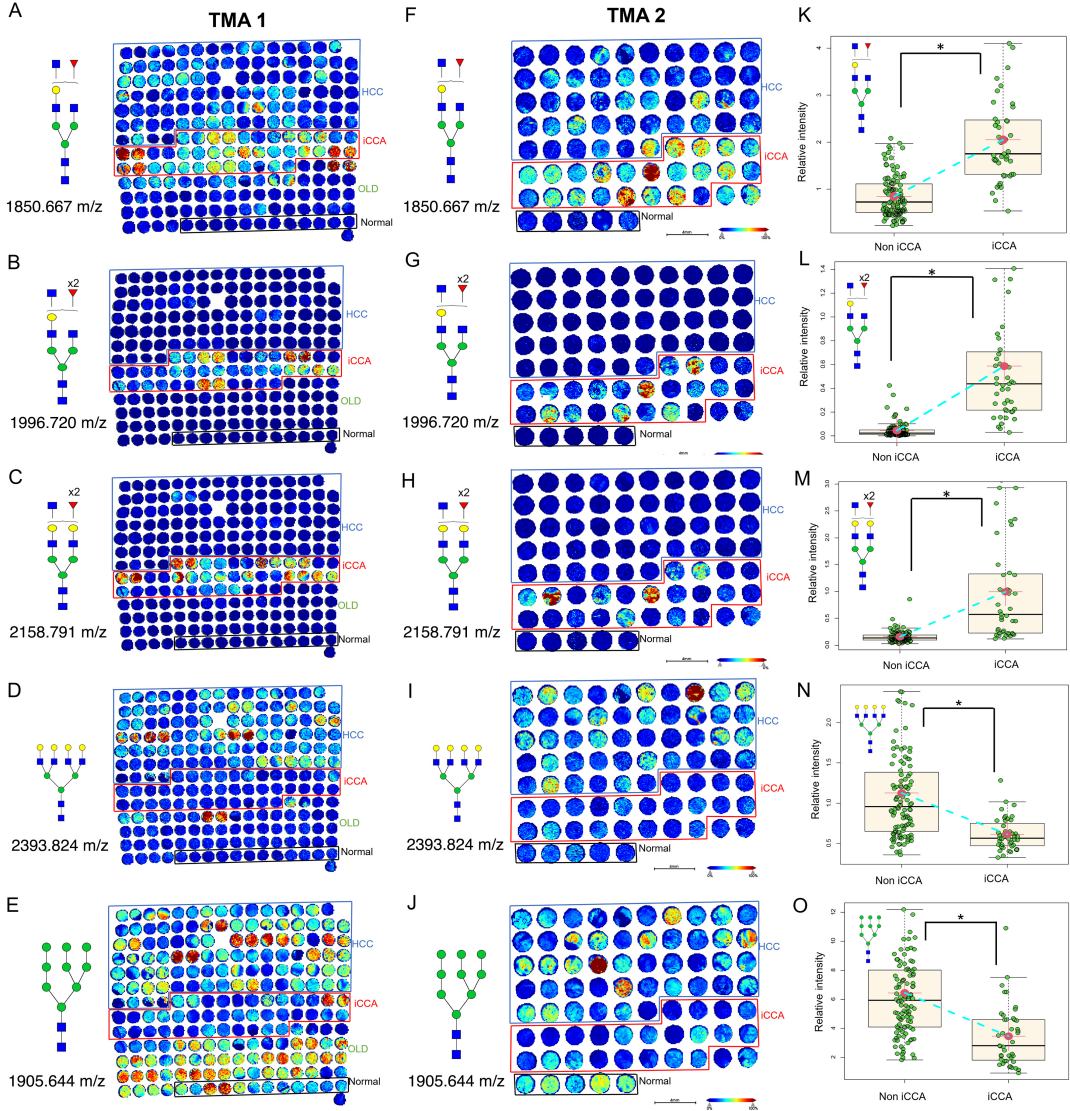
Bisected fucosylated N-glycan alterations are specific to patients with iCCA in tissue—analysis in a discovery and validation tissue cohort. Representative images of the relative intensity of bisected/triantennary single fucosylated N-glycan (1850.667 m/z); (**A**) double fucosylated N-glycan (1996.720 m/z); (**B**) double fucosylated with a galactose N-glycan (2158.791 m/z); (**C**) tetraantennary branched N-glycan (2393.824 m/z) N-glycan (**D**) and high mannose N-glycan (1905.644 m/z) in TMA 1 (**E**). This TMA includes two cores per patient: 50 HCC, 20 iCCA, and 6 normal hepatic tissue. OLD: other liver diseases. (**F–J**) same N-glycans as in **A**–**E** but analyzed in a second independent validation tissue cohort (TMA 2). This TMA includes one core per patient: 45 HCC, 23 iCCA, and 5 normal hepatic tissue. (**K–O**) relative intensity quantification of both TMAs comparing iCCA (*n* = 43) v non iCCA (*n* = 108). Each point in box plots represents a patient. The mass defect used for each m/z value is based on TMA 1 run. The asterisk indicates statistical difference (Mann–Whitney, *P* < 0.001) and error bars represent the SD. Darker red colors represent a higher intensity for the specific glycan while more blue tones represent less intensity. For N-glycans, red triangle, fucose; blue square, N-acetylglucosamine; green circles, mannose; yellow circles, galactose.

To determine whether the N-glycan alterations previously identified in iCCA tissue could be observed in a larger set of tissue samples, we examined a discovery TMA consisting of a total of 104 tissue cores: fifty HCC, twenty iCCA, one clear-cell carcinoma cyst, five metastatic HCC (spleen, chest wall, cerebrum, costal bone, and lymph node), two hepatic cysts, eight tissues with cirrhosis and dysplastic nodules, ten hepatitis infected, two adjacent normal tissues, and six independent normal tissues. Figure 2A–E shows representative images from this discovery TMA (TMA 1) demonstrating the N-glycan changes from MALDI-IMS N-glycan imaging data. Specific N-glycans at m/z values of 1850.667 (presumed bisected N-glycan with one fucose residue), 1996.720 (presumed bisected N-glycan with two fucose residues), 2158.791 (presumed bisected N-glycan with two fucose residues and with an additional galactose residue), 2012.717 (presumed bisected N-glycan with one fucose residue), and 1809.646 (biantennary N-glycan with one fucose residue) were found primarily in iCCA tissue (Fig. 2A–C; Supplementary Fig. S2D and S2E). In contrast, N-glycan at m/z 2393.824 was specific to HCC tissue when compared with iCCA or normal samples (Fig. 2D). In addition, N-glycan at 1905.644 followed a similar trend as in Fig. 1, where it was expressed in all groups with a decrease in the iCCA samples compared with other groups (Fig. 2E). Normal tissue was associated with high mannose N-glycans, and biantennary type N-glycan with and without the terminal sialic acid, with limited levels of fucosylation and branching, as we have observed before (15).

Next, we used a second TMA (TMA 2) for validation which consisted of a total of 75 tissue cores: forty-five HCC, twenty-three iCCA, two mixed HCC-iCCA, and five normal liver tissues (Fig. 2F–J). We identified the same N-glycans in our validation TMA as in our discovery of TMA in iCCA tissues. As before, bisected fucosylated N-glycans at m/z values of 1850.667, 1996.720, 2158.791, and 2012.717 are significantly increased in iCCA as compared with the other groups (Fig. 2F–H; Supplementary Fig. S2D), while N-glycans at 2393.824 and 1905.644 are either not elevated or underexpressed in iCCA (Fig. 2I–J). Each N-glycan alteration from the two TMAs was further analyzed by comparing their relative intensity in iCCA tissue with non iCCA tissue which included normal, and other liver diseases (described in TMA 1). As Fig. 2K–M show, bisected fucosylated N-glycans were significantly altered in the iCCA tissue (Fig. 2K–M) while complex highly branched and high mannose N-glycans were significantly decreased in iCCA tissue (Fig. 2N–O). While only N-glycan changes between iCCA, HCC, and normal tissues are detailed here, Supplementary Fig. S2E details N-glycan changes in other types of liver diseases present in TMA 1.

### Bisected Core Fucosylated N-glycan Alterations are Specific to Patients with iCCA in Tissue

**FIGURE 3:**
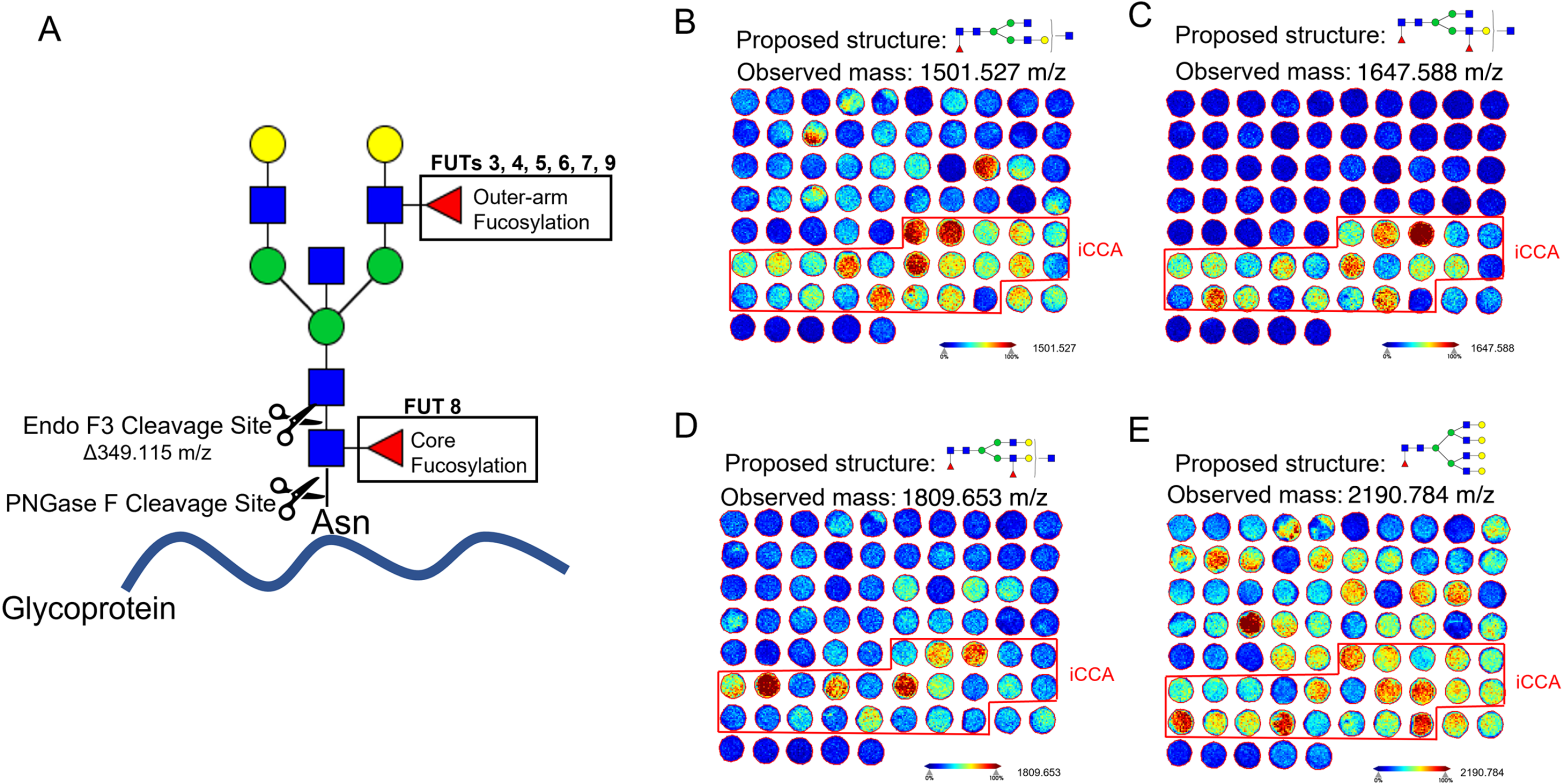
Bisected core fucosylated N-glycan alterations are specific to patients with iCCA in tissue. (**A**) Cartoon description of Endo F3 (including mass shift) and PNGase F cleavage sites, and the respective FUTs that catalyze the addition of the fucose residue. Representative images of core fucosylated N-glycans after Endo F3 treatment on TMA2, bisected core fucosylated N-glycan (**B**) bisected core, and outer-arm fucosylation N-glycan (**C**) bisected core, and outer-arm fucosylation with two galactose residues N-glycan (**D**) and tetraantennary core fucosylated N-glycan (**E**). For N-glycans, red triangle, fucose; blue square, N-acetylglucosamine; green circles, mannose; yellow circles, galactose.

TMA 1 and TMA 2 representative images from two different bisected N-glycans each with two fucose residues (Fig. 2B, C, G, and H) demonstrated a higher specificity to iCCA tissues relative to Fig. 2A and F which is also a bisected N-glycan with only one fucose residue. On the basis of this observation, we were interested to elucidate the origin of this fucosylation, that is core fucosylation or outer-arm fucosylation (Fig. 3A). We hypothesized that the specificity of bisected double fucosylated N-glycans in iCCA tissues was most likely due to core fucosylation after FUT8 and core fucosylation have been the most common modifications reported in cancer (14). Figure 3B–D demonstrates a higher intensity and specificity of core fucosylated N-glycans to iCCA tissues in bisected N-glycans, suggesting that the fucosylation previously observed is core fucosylation. While a fucosylated tetraantennary branched N-glycan (at 2190 m/z) had a high intensity of core fucosylated N-glycans, this was not specific to any of the groups (Fig. 3E), confirming that the specificity of these N-glycan structures to iCCA samples is due to the combination of bisected and core fucosylated N-glycans.

### Characterization of N-glycosylation Alterations in Serum of Patients with iCCA

**FIGURE 4:**
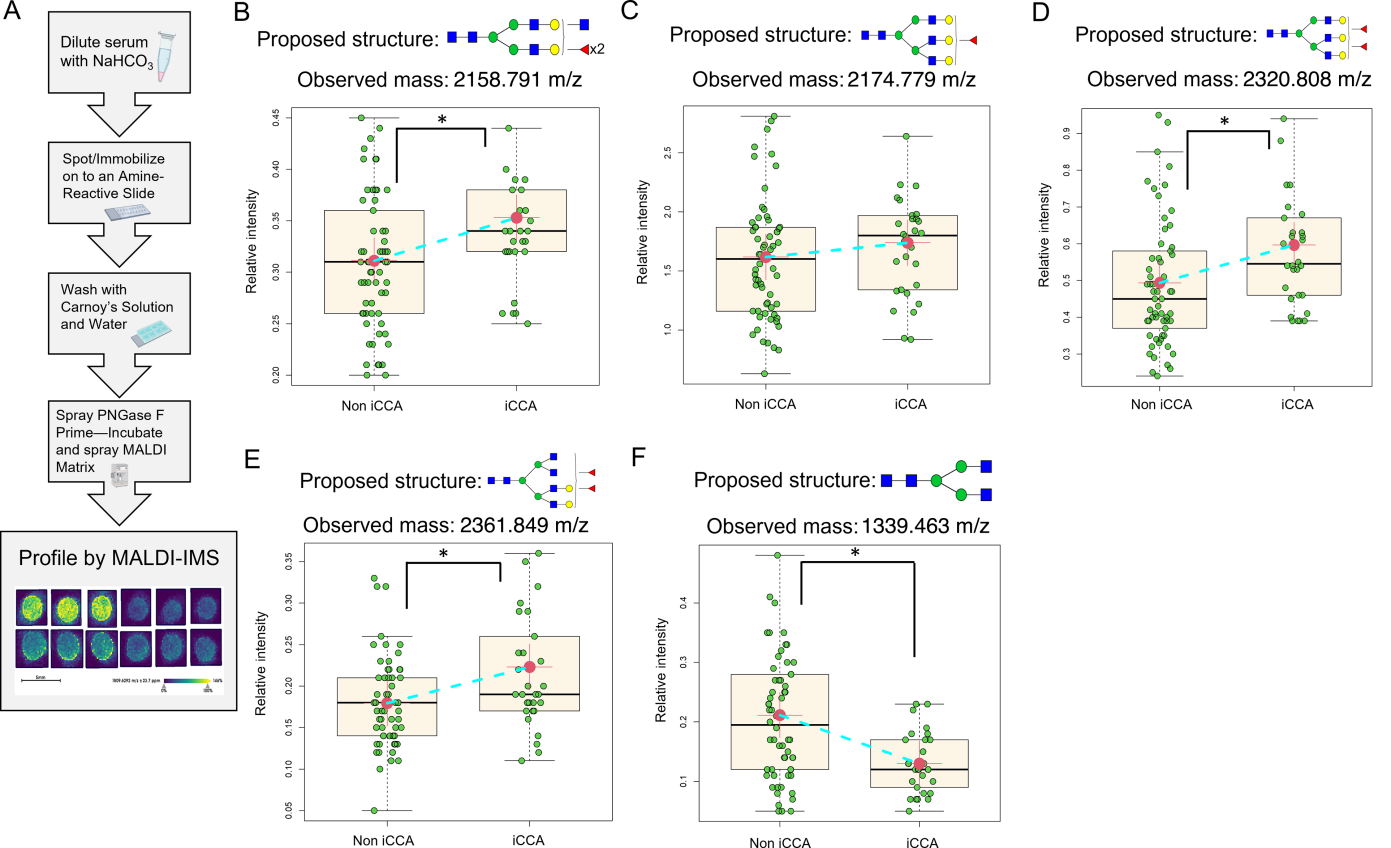
Fucosylated N-glycans are significantly altered in iCCA serum. (**A**) Serum N-glycan imaging workflow. Relative intensity quantification of bisected double fucosylated N-glycan (2158.791 m/z); (**B**) triantennary fucosylated N-glycan (2174.779 m/z); (**C**) triantennary double fucosylated N-glycan (2320.808 m/z); (**D**) and tetraantennary double fucosylated N-glycan (2361.849 m/z); (**E**) biantennary N-glycan (1339.463 m/z); (**F**) Each point in box plots represents a patient. The asterisk indicates statistical difference (Mann–Whitney, *P* < 0.001) and error bars represent the SD. Non iCCA *n* = 62 and iCCA *n* = 30. The mass defect used for each m/z value is based on TMA 1 run. For N-glycans, red triangle, fucose; blue square, N-acetylglucosamine; green circles, mannose; yellow circles, galactose.

To determine the translational potential of the N-glycan modifications seen in tissue to serum assays, we utilized a MALDI-IMS serum N-glycan profiling (28)**.**Figure 4A outlines the workflow used to process serum samples. Serum imaging data were analyzed on the basis of the relative intensity of each N-glycan and used for sample comparison between non iCCA (samples from patients at risk of developing CCA or other types of liver disease including HCC, cirrhosis, hepatitis, PSC, and fatty liver diseases) and patients with iCCA. Consistent with the trend observed in the discovery and validation TMAs, we found that the N-glycan bisected double fucosylated was significantly altered in this independent serum cohort (Fig. 4B). Similarly, other fucosylated (in this case triantennary and tetraantennary) N-glycans (2174 m/z, 2320 m/z, and 2361 m/z) were also significantly altered in the iCCA cohort compared with non iCCA (Fig. 4C–E). In addition, we identified N-glycan at 1339.463 m/z, a nonfucosylated bisected N-glycan (known as the core structure of an N-glycan structure) to be significantly decreased in the iCCA tissues (Fig. 4F). Overall, serum analysis revealed a very similar trend to what was observed in tissue, where bisected fucosylated and other fucosylated N-glycans continue to be highly altered in iCCA compared with any other group.

### Association of N-glycan Modifications Identified in Tissue to the Serum of Patients with iCCA

**FIGURE 5:**
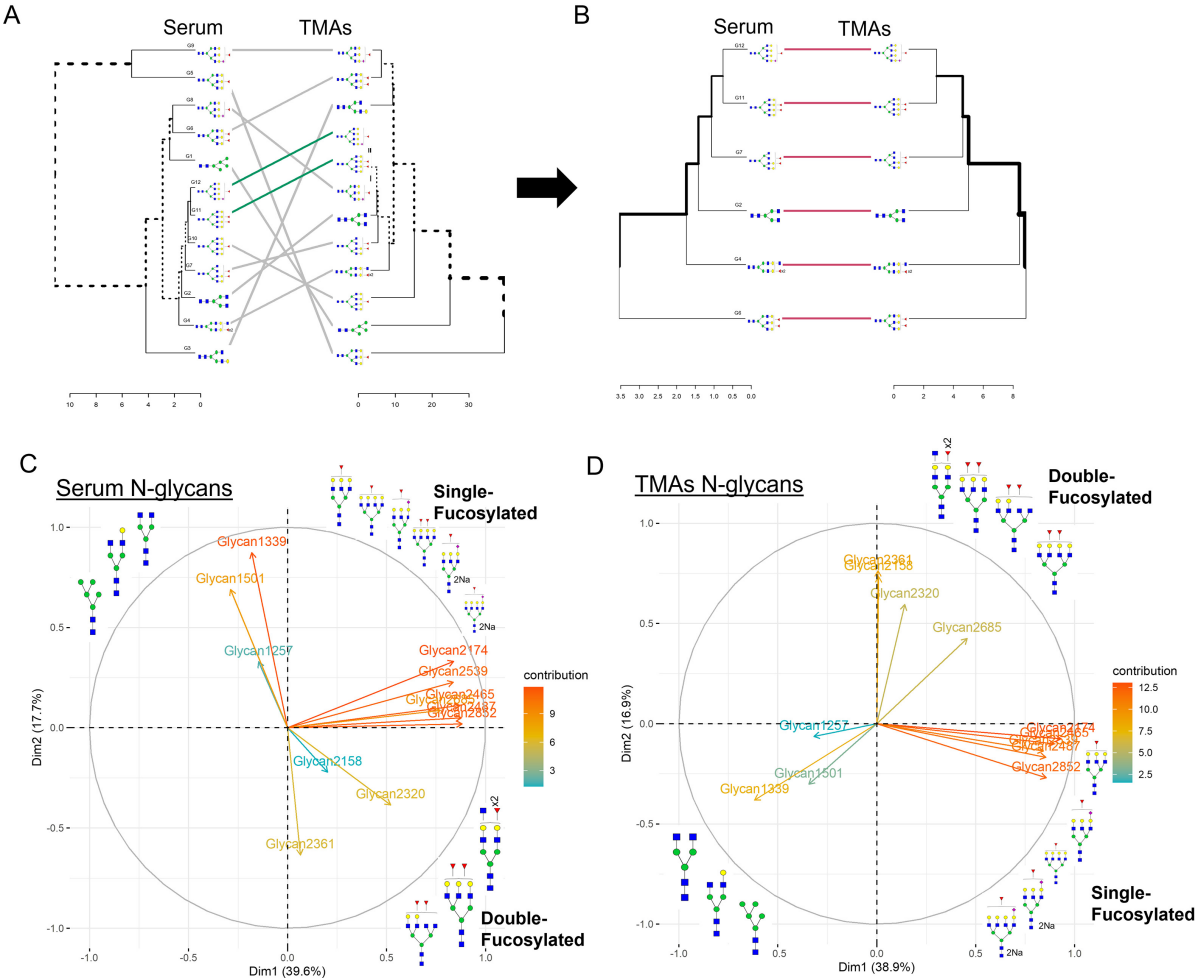
Profile data clustering reveals N-glycan grouping based on the number of fucose residues in iCCA serum and tissue. Dendrograms representing clustering for 12 N-glycans (**A**) and 6 N-glycans (**B**) in serum and TMAs. Glycan (G), G1: 1217, G2: 1339, G3:1501, G4:2158, G5:2174, G6:2320, G7:2631, G8:2465, G9:2487, G10:2539, G11:2685, G12:2852. PCA and their dimension (Dim) of serum (**C**) and TMAs (**D**) showing N-glycan clustering based on the number of fucose residues. 2Na (doubly sodiated N-glycan). For N-glycans, red triangle, fucose; blue square, N-acetylglucosamine; green circles, mannose; yellow circles, galactose.

Next, we performed a comprehensive analysis of all the N-glycan modifications observed in tissue and serum to determine the association between N-glycans found in tissue and serum. As before, we used the relative intensity of each N-glycan for the respective dataset (discovery TMA and validation TMA were analyzed and are referred to here as TMAs). Analysis of TMAs and serum revealed a total of 12 N-glycans that followed the same trend (upregulation or downregulation) with a significant *P* value in serum, TMA, or both for some N-glycans (Table 1). Next, these N-glycans were analyzed by their data structure with clustering analysis (Fig. 5A) and found that six specific N-glycans had a very similar data structure (Fig. 5B), where most of these six N-glycans were fucosylated with different N-glycan types: biantennary, bisecting, triantennary, and tetraantennary N-glycans (Fig. 5B). We continued our analysis using the initial 12 N-glycans identified to determine their data structure based on variability (Entropy) by PCA. Figure 5C for serum and Fig. 5D for TMAs, show that N-glycans profile cluster into three specific types based on the level of fucosylation (first, biantennary, not fucosylated; second, single fucosylated; and third, double fucosylated). Biplot of first principal component (dimension 1; accounts for 39% of the total variation in TMAs and serum datasets) and second principal component (dimension 2; accounts for 17% of the total variation in TMAs and serum datasets). Overall, double fucosylated N-glycans demonstrated higher informative contributions in the dataset. Supplementary Figure S3A specifies the relative contribution per glycan to the first and second principal components indicated in plots with their relative contribution per N-glycan.

**TABLE 1:**
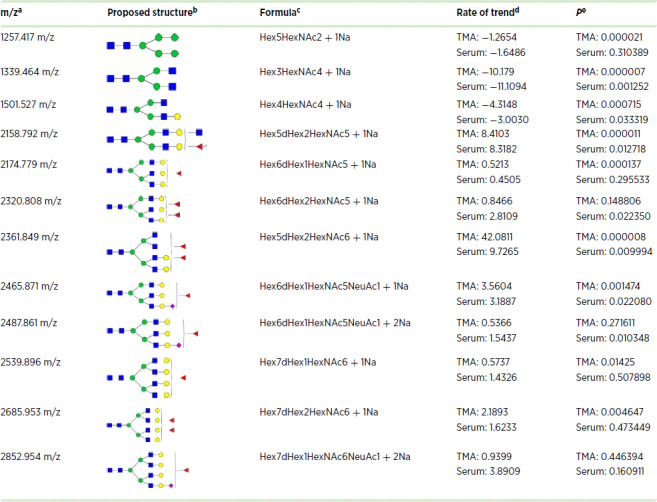
Identified N-glycans with the same trend between TMA and serum

NOTE: For N-glycans, red triangle, fucose; blue square, N-acetylglucosamine; green circles, mannose; yellow circles, galactose.

Abbreviation: TMA, tissue microarray.

^a^Observed mass-to-charge ratio (m/z) value.

^b^The proposed glycan structure based upon the m/z value.

^c^Composition of the identified m/z value.

^d^Rate of trend in TMA and serum datasets.

^e^The *P* value in TMA and serum datasets.

**FIGURE 6:**
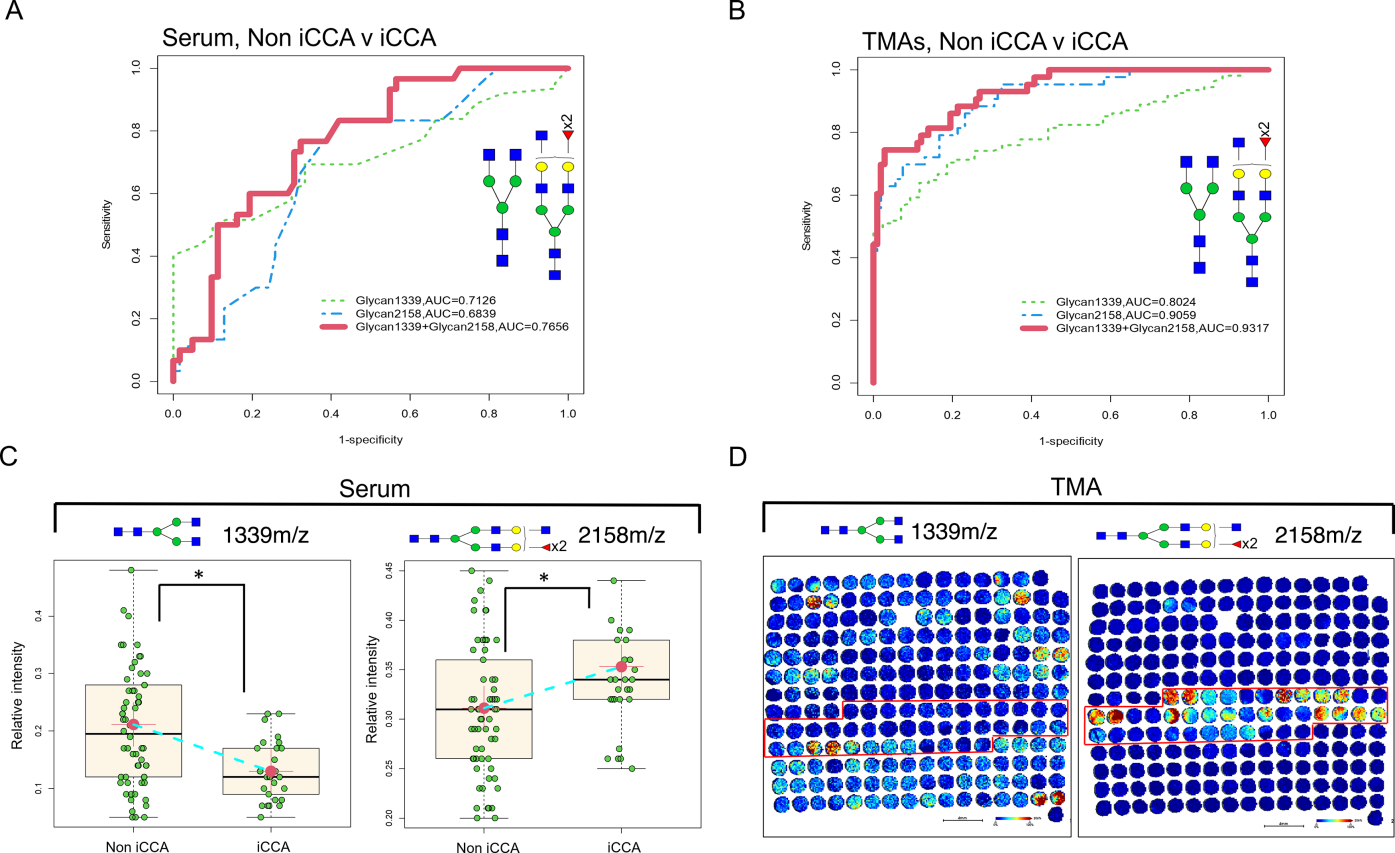
N-glycans from serum and TMA as promising biomarkers to differentiate iCCA from other liver diseases. (**A**) ROC curve for serum. Non iCCA *n* = 62 and iCCA *n* = 30. (**B**) ROC curve for TMA, non iCCA *n* = 108 and iCCA *n* = 43. For ROC curve labeling: N-glycan 1339 m/z (green-dashed line), N-glycan 2158 m/z (blue-dashed line), and a combination of N-glycans 1339 m/z and 2158 m/z (red-solid line). (**C**) Relative intensity quantification boxplots of N-glycan 1339 m/z and 2158 m/z in serum, non iCCA *n* = 62, and iCCA *n* = 30. (**D**) Representative images of TMA 1 showing the N-glycan intensity for 1339 m/z and 2158 m/z. The asterisk indicates statistical difference (Mann–Whitney, *P* < 0.001) and error bars represent the SD. For N-glycans, red triangle, fucose; blue square, N-acetylglucosamine; green circles, mannose; yellow circles, galactose.

To identify specific N-glycans that could distinguish between patients with iCCA and non iCCA, we optimized the 12 N-glycans, this revealed three main N-glycans per dataset (Supplementary Fig. S3B). In the TMAs analysis, the N-glycans were at 1339 m/z, 1257 m/z (high mannose N-glycan), and 2158 m/z (Supplementary Fig. S3B). For the serum analysis, the N-glycans were a 1339 m/z, 2158 m/z, and 2361 m/z (highly branched tetraantennary, double fucosylated; Supplementary Fig. S3B). In addition, we investigated if the different types of iCCA (small duct and large duct) could express a different N-glycan profile, and found that there were no significant differences in any of these glycans, suggesting that these N-glycans are independent of the type of iCCA (Supplementary Fig. S3C). From these three main N-glycans, only the two common N-glycans (1339 m/z and 2158 m/z) between tissue and serum were further analyzed (Supplementary Fig. S3D). The ROC curve from common N-glycans combined in serum had an AUC of 0.7656 (Fig. 6A); while these same common N-glycans in the TMAs had an AUC of 0.9317 (Fig. 6B). Interestingly, N-glycan 1339 m/z and 2158 m/z had an opposing trend in serum (Fig. 6C) and tissue (Fig. 6D), where 1339 m/z was significantly decreased in iCCA while 2158 m/z was significantly increased. This analysis suggests that the use of bisecting fucosylated N-glycans and the core N-glycan structure have a powerful discrimination ability that could be a promising differentiator strategy between patients with iCCA and non iCCA.

Next, we explored further the importance of 1339 m/z as one of the differentiators between iCCA and non iCCA in serum because this N-glycan modification has not been associated with liver cancer before to our knowledge. We excluded this N-glycan from the 12 N-glycans from our analysis and performed a feature selection from a random forest algorithm. We identified, 5 N-glycans: 2465 m/z and 2487 m/z (triantennary fucosylated with a sialic acid residue and same structure sulfated, respectively), 2158 m/z, 2320 m/z, and 2174 m/z. Supplementary Figure S3E shows the importance of each N-glycan selected, with all N-glycans being bisected/triantennary fucosylated. Combining these N-glycans to determine their ability to distinguish between iCCA and non iCCA resulted in an AUC of 1 for classification and an AUC of 0.7151 for LOOCV (Supplementary Fig. S3F). This analysis demonstrates the importance of N-glycan 1339 m/z in our model because its exclusion resulted in the need for 5 N-glycans to compensate for the use as a powerful discriminator (Supplementary Fig. S3F). However, this still confirms our previous analysis in which fucosylated bisected and triantennary structures have important roles in iCCA.

**FIGURE 7:**
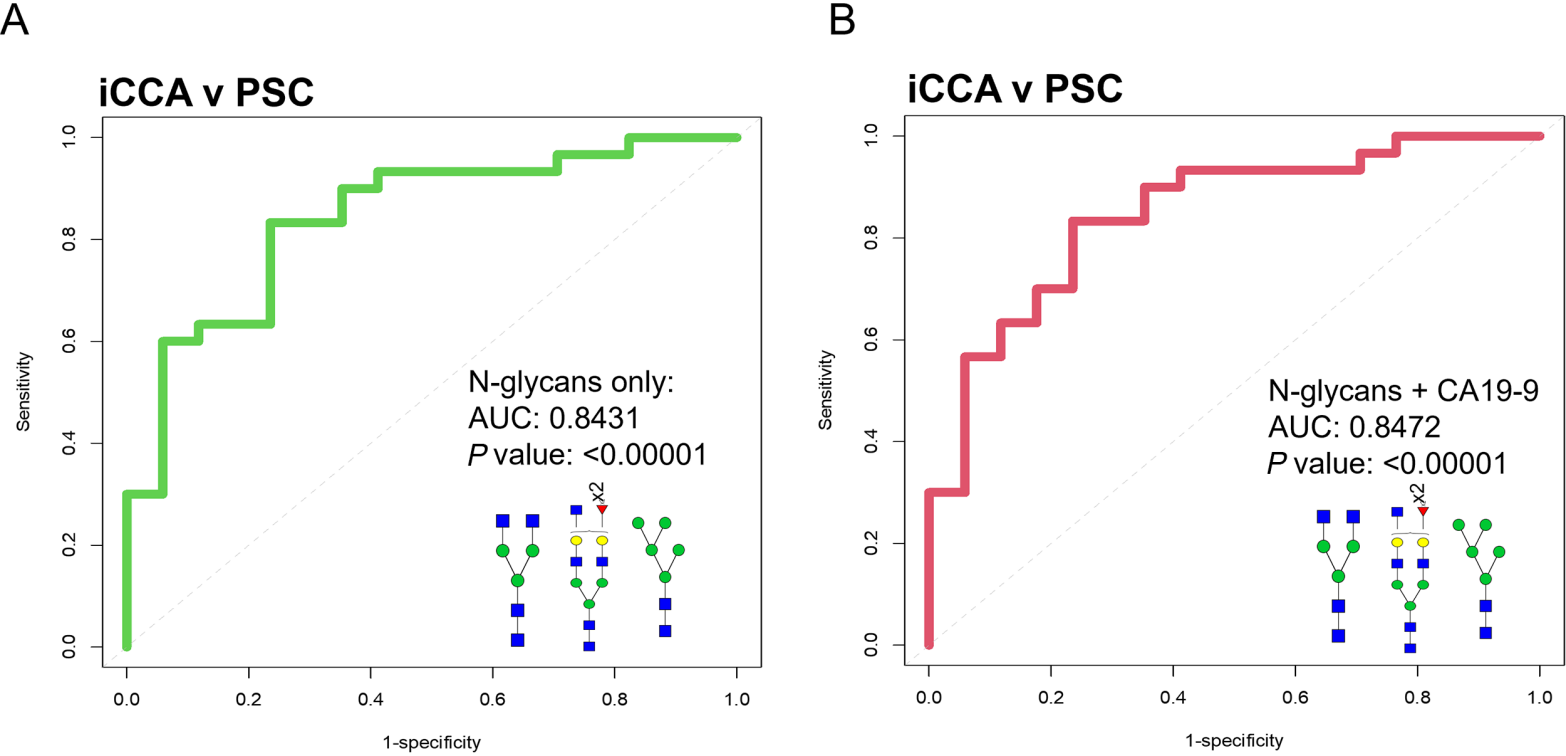
N-glycan combinations as promising biomarkers to differentiate iCCA from PSC. (**A**) ROC curve classification of a combination of 1339 m/z, 2158 m/z, and 1257 m/z. (**B**) ROC curve of combination of 1339 m/z, 2158 m/z, 1257 m/z, and CA19-9. For ROC curves: iCCA (*n* = 30) and PSC (*n* = 17). For N-glycans, red triangle, fucose; blue square, N-acetylglucosamine; green circles, mannose; yellow circles, galactose.

Finally, to implement a clinical translational aspect for biomarker discovery using the N-glycan molecular changes presented here, we added CA19-9 information to our analysis. Serum CA19-9 is a biomarker used to identify those who might require diagnostic imaging for CCA. For this analysis, we focused on patients for whom we had CA19-9 information available (30 patients with iCCA and 17 patients with PSC). Strategies for the diagnosis of iCCA in PSC are urgently needed because early detection of iCCA can improve patients’ survival and the conventional strategies share many features between conditions making diagnosis unsuccessful in most patients (3). Supplementary Table S2 lists the small capability of individual glycans (AUC: 0.500–0.727), CA19-9 (AUC: 0.512), age (AUC: 0.654), or liver enzymes (AUC: 0.558–0.697) to differentiate between patients with iCCA or PSC. However, when we combined some of the most significant N-glycans (1339, 1257, and 2158), this resulted in a significant ability of this combination to differentiate between patients with PSC to those with iCCA (AUC: 0.8431, *P* ≤ 0.00001; Fig. 7A; Supplementary Fig. S3G). We demonstrate this is independent of CA19-9 because including CA19-9 information in the N-glycan combination, did not significantly improve biomarker performance (AUC: 0.8472; Fig. 7B; Supplementary Fig. S3G). Finally, an additional multivariate model on N-glycans 1339, 1257, and 2158 and the clinical information available for patients with PSC and iCCA revealed that the ability of these N-glycan to differentiate between diagnosis is independent of the clinical information applied (Supplementary Fig. S4A–C). A complete N-glycan profile quantification between the serum and tissue is presented in Supplementary Fig. S5A. Overall, we demonstrate that the combination of specific N-glycan modifications can be a promising biomarker for identifying patients with iCCA from those with PSC.

## Discussion

Alterations in N-glycosylation have been long observed with HCC (18, 21, 34–36), and we and others have shown that increased levels of some fucosylated glycoproteins could be observed in the serum of patients with CCA (7–9, 37). However, a study that elucidates the origin of these N-glycan modifications while also exploring the same modifications in iCCA serum as a possible biomarker has not been done before.

In this study, we identified bisecting, branching (triantennary), and fucosylation as specific N-glycan structure modifications in iCCA tissue and serum. These N-glycan modifications are known to be catalyzed by the following glycosyltransferases: First, β-1,4-mannosyl-glycoprotein 4-beta-N-acetylglucosaminyltransferase (MGAT3) for bisecting, which has been previously reported to differentiate between iCCA and HCC in serum (38). In addition, MGAT3 has been considered a malignancy suppressor where its overexpression can inhibit metastatic profiles of cancer cells (39). Second, α-1,6-mannosylglycoprotein 6-beta-N-acetylglucosaminyltransferase A (MGAT5) for branching, which has been linked to malignancy and correlates with disease progression (40). In addition, its activation in many cancers has been reported to be through the upregulation of the RAS-RAF-MAPK signaling pathway (39). Finally, another major alteration observed in the patients with CCA was increased fucosylation. We and others have shown that core fucosylation, catalyzed by alpha-1,6-fucosyltransferase (FUT8), is one of the main N-glycan modifications in early liver disease like nonalcoholic fatty liver disease and liver cancers (7, 41–43). FUT8 expression has also been shown to increase as cells undergo an epithelial–mesenchymal transition by remodeling core fucosylation on the TGFβ receptor (44). Previous studies in CCA serum have reported increased double fucosylation with triantennary N-glycans (7, 8, 37). Similarly, we demonstrate here that alterations in fucosylation are due to outer-arm and core fucosylation, but core fucosylation had a higher specificity to iCCA in tissue (Fig. 3B–D).

This study elucidates N-glycan modifications that can be specific to each type of liver cancer in tissue. Patients with iCCA had a very specific expression of 2158 m/z (presumed bisected double fucosylated N-glycan), which was not present in HCC or normal samples (Fig. 2C, H, and M). In contrast, only patients with HCC expressed 2393 m/z (tetraantennary branched N-glycan), which was not present in iCCA and/or normal samples (Fig. 2D, I, and N). This suggests N-glycosyltransferases MGAT 3 and MGAT 5 have different roles in cancer progression according to the type of liver cancer. The changes in specific N-glycan structures according to the type of cancer observed here can be explained by the opposing roles of MGAT3 and MGAT5. MGAT3-generated bisecting structures are less readily bound and modified by MGAT5, resulting in a decrease in triantennary and tetraantennary structures. Accordingly, increased intensity of bisecting structures correlated with decreased intensity of tetraantennary structures in patients with iCCA, but patients with HCC had no changes in bisecting structures and increased highly branched structures. Future studies should investigate the exact roles of MGAT3 and MGAT5 in the context of each type of liver cancer.

An interesting N-glycan modification that has not previously been linked to iCCA was 1339 m/z (biantennary nonfucosylated N-glycan) which in this study had a decreased intensity in iCCA tissue and serum. Statistical analysis revealed the importance of this modification when coupled with bisected fucosylated structures as a biomarker candidate for differentiating between iCCA v non-iCCA and iCCA v PSC. We hypothesize that the reduction in the expression of this N-glycan is due to this structure being modified to higher/complex N-glycan structures because this is the basic core of an N-glycan structure.

A translational point of this study was correlating tissue and serum analysis for the characterization of N-glycan–related molecular changes because most of the serum N-glycoproteins are synthesized by the hepatobiliary system and can reflect liver health. Utilizing N-glycan alterations that were correlated in tissue and serum analysis improved biomarker detection and should be pursued for the investigation of other diseases, especially those impacting the liver. It is important to note that not all N-glycans followed the same trend from tissue to serum. We hypothesize these inconsistencies may be due to the large contribution of IgG to the serum N-glycan profile.

Here, we utilized MALDI-IMS N-glycan imaging to identify the modifications that occur directly in iCCA patient tissue and serum samples. In our whole tissue analysis, we elucidate the histopathologic origin of N-glycan structures in the iCCA and HCC tissue. Next, we analyzed two tissue TMA cohorts (a discovery and validation cohort) and an independent serum cohort. Finally, we used tissue (from discovery and validation cohorts) and serum N-glycan alterations and identified 2158 m/z (presumed bisecting double fucosylated) and 1339 m/z (biantennary nonfucosylated) as the most effective combination to distinguish between iCCA and non iCCA in tissue and serum. While 1339 m/z, 2158 m/z, and 1257 m/z (high mannose N-glycan) as biomarker candidates to distinguish between patients with iCCA and PSC in serum.

Together, the data presented here suggest that the changes characterized here in tissue and serum could originate from cancer itself and prior analytic glycan tools were not able to detect these changes within the tissue. A matched serum and tissue cohort would be needed to confirm the origin of these N-glycan alterations. To conclude, we propose the use of N-glycan alterations as promising biomarkers for their ability to differentiate between intrahepatic iCCA, HCC, PSC, and benign chronic liver disease. Further studies should be done using a larger set of serum samples to validate the value of the biomarker candidates proposed here.

## Supplementary Material

Supplementary Figure SF1 Hematoxylin & Eosin (H&E) staining of A. Intrahepatic Cholangiocarcinoma (iCCA) tissue, 10x (right) and 40x (left) magnification images from respective areas of the tissue, and B. Hepatocellular Carcinoma (HCC) tissue, 10x magnification images from respective areas of the tissue. Tumor regions are outlined in red, normal areas are outlined in black and fibrotic regions are outlined in blue. C. TMA H&E staining with an outline that specifies the diagnosis for each core for TMA 1 (left) and TMA 2 (right). Mixed carcinoma: HCC and iCCA. Small (yellow) and large (purple) ducts classifications for each TMA.

Supplementary Figure SF2 Patient demographics for A. TMA 1 and B. TMA 2. C. Patient demographics for serum cohort. ALT (Alanine transaminase), AST (aspartate aminotransferase), AFP (Alpha-fetoprotein), and ALP (Alkaline phosphatase). Other liver diseases include nonalcoholic steatohepatitis, hepatitis C with cirrhosis, hepatic adenoma, benign fibrotic gallbladder disease, and diabetes. PSC (Primary Sclerosing cholangitis), HCC (Hepatocellular carcinoma), and OLD (Other liver diseases). Gray shading for missing clinical information. D. Representative N-glycan images of TMA 1 (top) and TMA 2 (bottom) of 2012.717m/z (left) and 1809.646m/z (right). Red boxes select for CCA samples. E. Table details other pathology diagnoses included in TMA 1 with the proposed structure for the N-glycans highly expressed in each. These modifications are characterized based on 1-2 patients.

Supplementary Figure SF3 A. Relative contribution of each serum-glycan (left) and TMA-glycan (right) in the first and second principal components. The size and color of the circle represent a higher contribution of the glycan to the respective Dim. (Dimension). B. Three N-glycans in TMA and serum were identified after optimization. C. Relative intensity quantification for both TMAs of the respective N-glycan based on small and large duct classification. D. Two common N-glycans were identified between datasets. LOOCV (Leave-One-Out Cross-Validation). AUC (Area Under the Curve). E. Quantification of the relative contribution of N-glycans (left), table of N-glycans, and proposed structure with importance values (top) when removing N-glycan at 1339 m/z from the analysis. F. ROC (Receiving Operator Characteristic) curve classification for serum (left) ROC curve LOOCV (right) of N-glycans in E. G. List combinations of N-glycan as possible biomarkers to differentiate iCCA from PSC. For N-glycans, red triangle, fucose; blue square, N-acetylglucosamine; green circles, mannose; yellow circles, galactose.

Supplementary Figure SF4 A. Scatterplots and correlations between N-glycans of interest (1339, 1257, 2158) and clinical information available (ALT, AST, ALK, and AFP). B. Multivariate model-Multiple logistic regression in CCA and PSC serum samples (n=40). Model 1: three N-glycans of interest and clinical information available (left panel). Model 2: Only the three N-glycans of interest (right panel). C. ROC curve of the combination of glycans and clinical information (red-solid line) and only glycans (green-dashed line) (left), classification performance table of the two models in B. p=0.5731, Delong’s test between model 1 and 2. CA19-9, ALT, AST, ALK, and AFP values were log-transformed for plotting and modeling convenience. logALT was removed from the multiple logistic regression analysis due to a high value of Variance inflation factor (VIF).

Supplementary Figure SF5 Relative intensity quantification of all N-glycans identified in serum (left row) and tissue (right row) analysis. Red font labeling for N-glycans follows the same trend between serum and tissue.

Supplementary Table ST1 Peak list of N-glycans detected in tissue (TMA) and serum samples.

Supplementary Table ST2 Lists individual N-glycans, ALT (Alanine transaminase), AST (aspartate aminotransferase), ALK (Alkaline phosphatase), and AFP (Alpha-fetoprotein) of analysis to differentiate patients with iCCA from PSC. 1Possible biomarkers 2n: number of total patients 3distribution of iCCA (n=30) and PSC (n=17) patients. 4AUC (Area Under the Curve) 5CI (confidence interval) 6p value. Some iCCA patients were excluded from the analysis due to missing clinical information.
