# Supplementary material for: Analysis of N-linked Glycan Alterations in Tissue and Serum Reveals Promising Biomarkers for Intrahepatic Cholangiocarcinoma
Source: Cancer Res Commun. 2023 Mar 6;3(3):383–94. doi: 10.1158/2767-9764.CRC-22-0422 (PMC9987250; doi:10.1158/2767-9764.CRC-22-0422)
Supplement: Supplementary Figure SF1 — Hematoxylin & Eosin (H&E) staining of A. Intrahepatic Cholangiocarcinoma (iCCA) tissue, 10x (right) and 40x (left) magnification images from respective areas of the tissue, and B. Hepatocellular Carcinoma (HCC) tissue, 10x magnification images from respective areas of the tissue. Tumor regions are outlined in red, normal areas are outlined in black and fibrotic regions are outlined in blue. C. TMA H&E staining with an outline that specifies the diagnosis for each core for TMA 1 (left) and TMA 2 (right). Mixed carcinoma: HCC and iCCA. Small (yellow) and large (purple) ducts classifications for each TMA. [file crc-22-0422-s01.docx]

**S1**


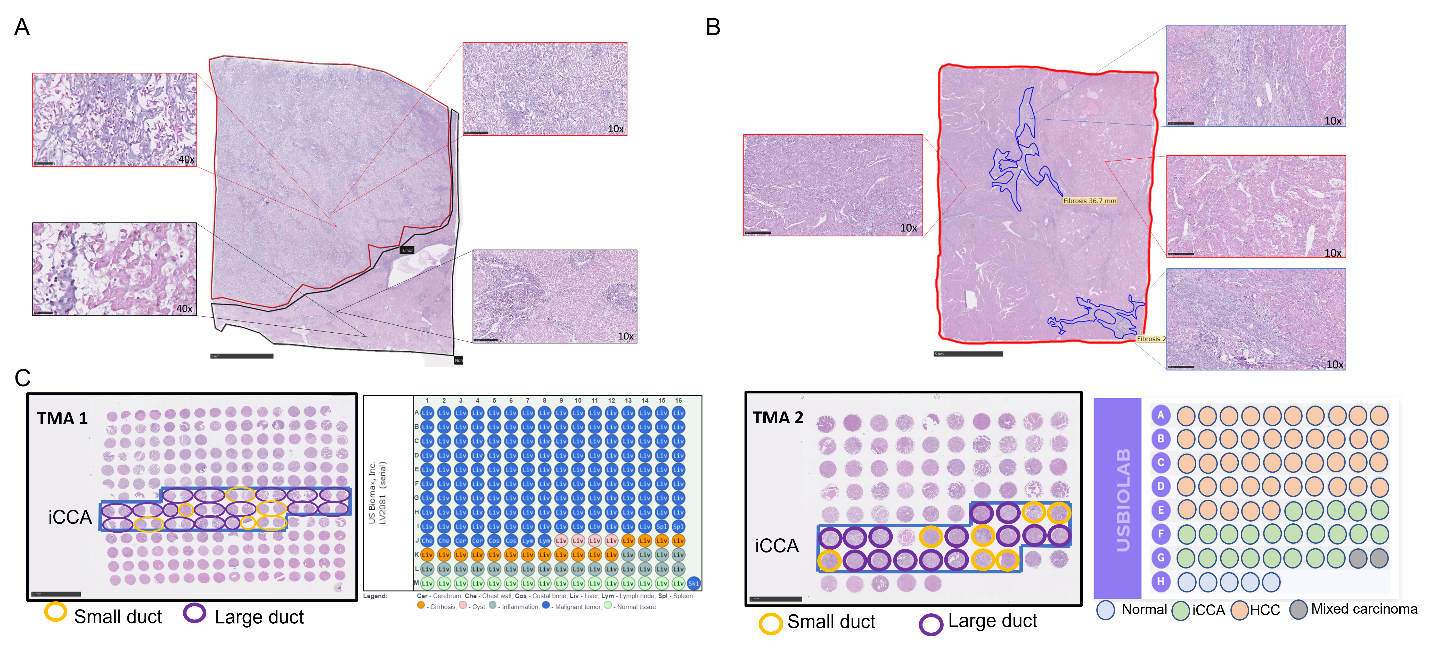


**Supplementary Figure 1.** Hematoxylin & Eosin (H&E) staining of **A.** Intrahepatic Cholangiocarcinoma (iCCA) tissue, 10x (right) and 40x (left) magnification images from respective areas of the tissue, and **B.** Hepatocellular Carcinoma (HCC) tissue, 10x magnification images from respective areas of the tissue. Tumor regions are outlined in red, normal areas are outlined in black and fibrotic regions are outlined in blue. **C.** TMA H&E staining with an outline that specifies the diagnosis for each core for TMA 1 (left) and TMA 2 (right). Mixed carcinoma: HCC and iCCA. Small (yellow) and large (purple) ducts classifications for each TMA.
