# Supplementary material for: Analysis of N-linked Glycan Alterations in Tissue and Serum Reveals Promising Biomarkers for Intrahepatic Cholangiocarcinoma
Source: Cancer Res Commun. 2023 Mar 6;3(3):383–94. doi: 10.1158/2767-9764.CRC-22-0422 (PMC9987250; doi:10.1158/2767-9764.CRC-22-0422)
Supplement: Supplementary Figure SF2 — Patient demographics for A. TMA 1 and B. TMA 2. C. Patient demographics for serum cohort. ALT (Alanine transaminase), AST (aspartate aminotransferase), AFP (Alpha-fetoprotein), and ALP (Alkaline phosphatase). Other liver diseases include nonalcoholic steatohepatitis, hepatitis C with cirrhosis, hepatic adenoma, benign fibrotic gallbladder disease, and diabetes. PSC (Primary Sclerosing cholangitis), HCC (Hepatocellular carcinoma), and OLD (Other liver diseases). Gray shading for missing clinical information. D. Representative N-glycan images of TMA 1 (top) and TMA 2 (bottom) of 2012.717m/z (left) and 1809.646m/z (right). Red boxes select for CCA samples. E. Table details other pathology diagnoses included in TMA 1 with the proposed structure for the N-glycans highly expressed in each. These modifications are characterized based on 1-2 patients. [file crc-22-0422-s02.docx]

**Supplementary Figure 2.** Patient demographics for **A.** TMA 1 and **B.** TMA 2. **C.** Patient demographics for serum cohort. ALT (Alanine transaminase), AST (aspartate aminotransferase), AFP (Alpha-fetoprotein), and ALP (Alkaline phosphatase). Other liver diseases include nonalcoholic steatohepatitis, hepatitis C with cirrhosis, hepatic adenoma, benign fibrotic gallbladder disease, and diabetes. PSC (Primary Sclerosing cholangitis), HCC (Hepatocellular carcinoma), and OLD (Other liver diseases). Gray shading for missing clinical information. **D.** Representative N-glycan images of TMA 1 (top) and TMA 2 (bottom) of 2012.717m/z (left) and 1809.646m/z (right). Red boxes select for CCA samples. **E.** Table details other pathology diagnoses included in TMA 1 with the proposed structure for the N-glycans highly expressed in each. These modifications are characterized based on 1-2 patients.
