# Supplementary material for: Analysis of N-linked Glycan Alterations in Tissue and Serum Reveals Promising Biomarkers for Intrahepatic Cholangiocarcinoma
Source: Cancer Res Commun. 2023 Mar 6;3(3):383–94. doi: 10.1158/2767-9764.CRC-22-0422 (PMC9987250; doi:10.1158/2767-9764.CRC-22-0422)
Supplement: Supplementary Figure SF3 — A. Relative contribution of each serum-glycan (left) and TMA-glycan (right) in the first and second principal components. The size and color of the circle represent a higher contribution of the glycan to the respective Dim. (Dimension). B. Three N-glycans in TMA and serum were identified after optimization. C. Relative intensity quantification for both TMAs of the respective N-glycan based on small and large duct classification. D. Two common N-glycans were identified between datasets. LOOCV (Leave-One-Out Cross-Validation). AUC (Area Under the Curve). E. Quantification of the relative contribution of N-glycans (left), table of N-glycans, and proposed structure with importance values (top) when removing N-glycan at 1339 m/z from the analysis. F. ROC (Receiving Operator Characteristic) curve classification for serum (left) ROC curve LOOCV (right) of N-glycans in E. G. List combinations of N-glycan as possible biomarkers to differentiate iCCA from PSC. For N-glycans, red triangle, fucose; blue square, N-acetylglucosamine; green circles, mannose; yellow circles, galactose. [file crc-22-0422-s03.docx]

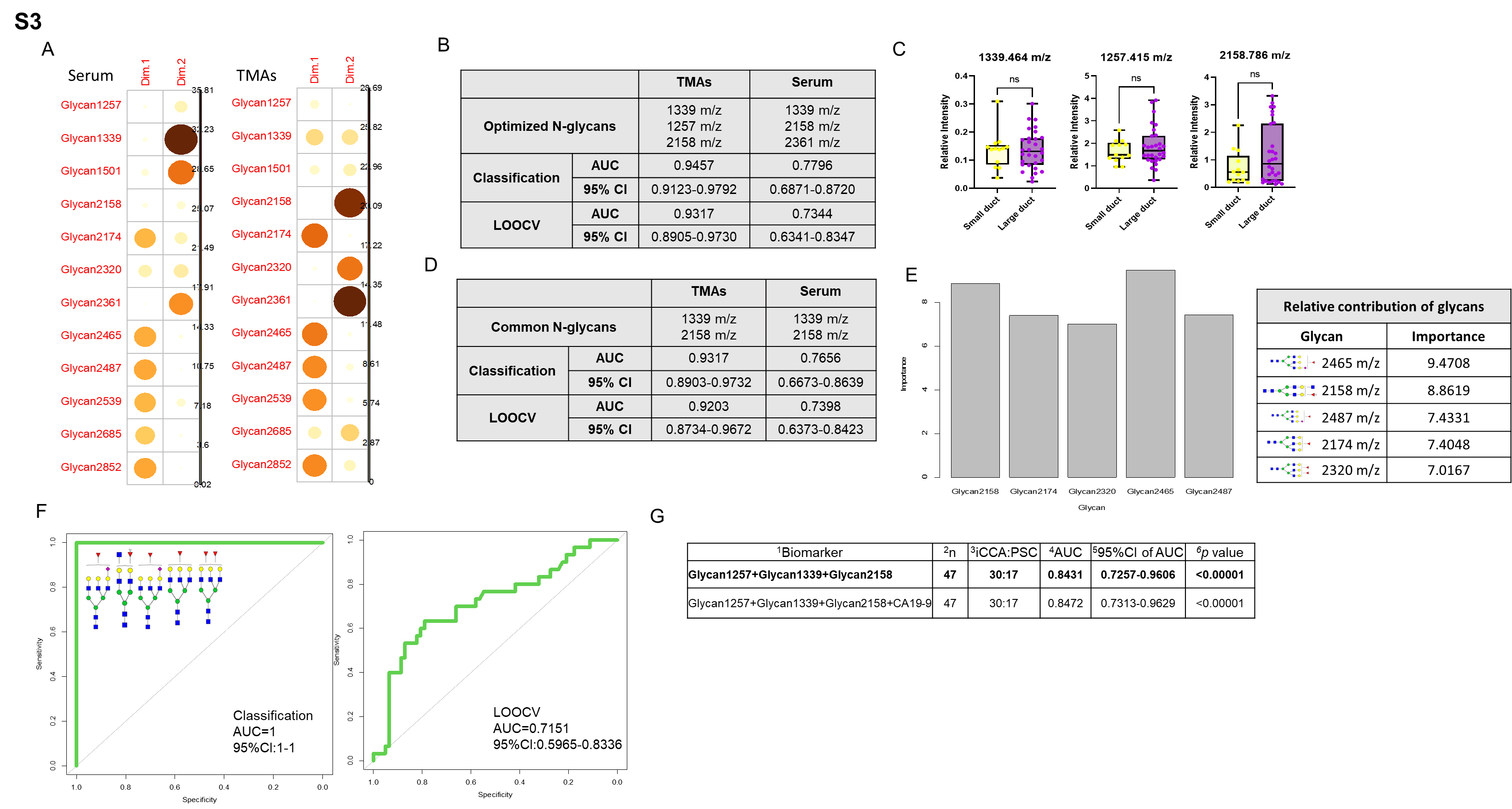


**Supplementary Figure 3. A.** Relative contribution of each serum-glycan (left) and TMA-glycan (right) in the first and second principal components. The size and color of the circle represent a higher contribution of the glycan to the respective Dim. (Dimension). **B.** Three N-glycans in TMA and serum were identified after optimization. **C.** Relative intensity quantification for both TMAs of the respective N-glycan based on small and large duct classification. **D.** Two common N-glycans were identified between datasets. LOOCV (Leave-One-Out Cross-Validation). AUC (Area Under the Curve). **E.** Quantification of the relative contribution of N-glycans (left), table of N-glycans, and proposed structure with importance values (top) when removing N-glycan at 1339 m/z from the analysis. **F.** ROC (Receiving Operator Characteristic) curve classification for serum (left) ROC curve LOOCV (right) of N-glycans in E. **G.** List combinations of N-glycan as possible biomarkers to differentiate iCCA from PSC. For N-glycans, red triangle, fucose; blue square, N-acetylglucosamine; green circles, mannose; yellow circles, galactose.
