# Supplementary material for: Analysis of N-linked Glycan Alterations in Tissue and Serum Reveals Promising Biomarkers for Intrahepatic Cholangiocarcinoma
Source: Cancer Res Commun. 2023 Mar 6;3(3):383–94. doi: 10.1158/2767-9764.CRC-22-0422 (PMC9987250; doi:10.1158/2767-9764.CRC-22-0422)
Supplement: Supplementary Figure SF4 — A. Scatterplots and correlations between N-glycans of interest (1339, 1257, 2158) and clinical information available (ALT, AST, ALK, and AFP). B. Multivariate model-Multiple logistic regression in CCA and PSC serum samples (n=40). Model 1: three N-glycans of interest and clinical information available (left panel). Model 2: Only the three N-glycans of interest (right panel). C. ROC curve of the combination of glycans and clinical information (red-solid line) and only glycans (green-dashed line) (left), classification performance table of the two models in B. p=0.5731, Delong’s test between model 1 and 2. CA19-9, ALT, AST, ALK, and AFP values were log-transformed for plotting and modeling convenience. logALT was removed from the multiple logistic regression analysis due to a high value of Variance inflation factor (VIF). [file crc-22-0422-s04.docx]

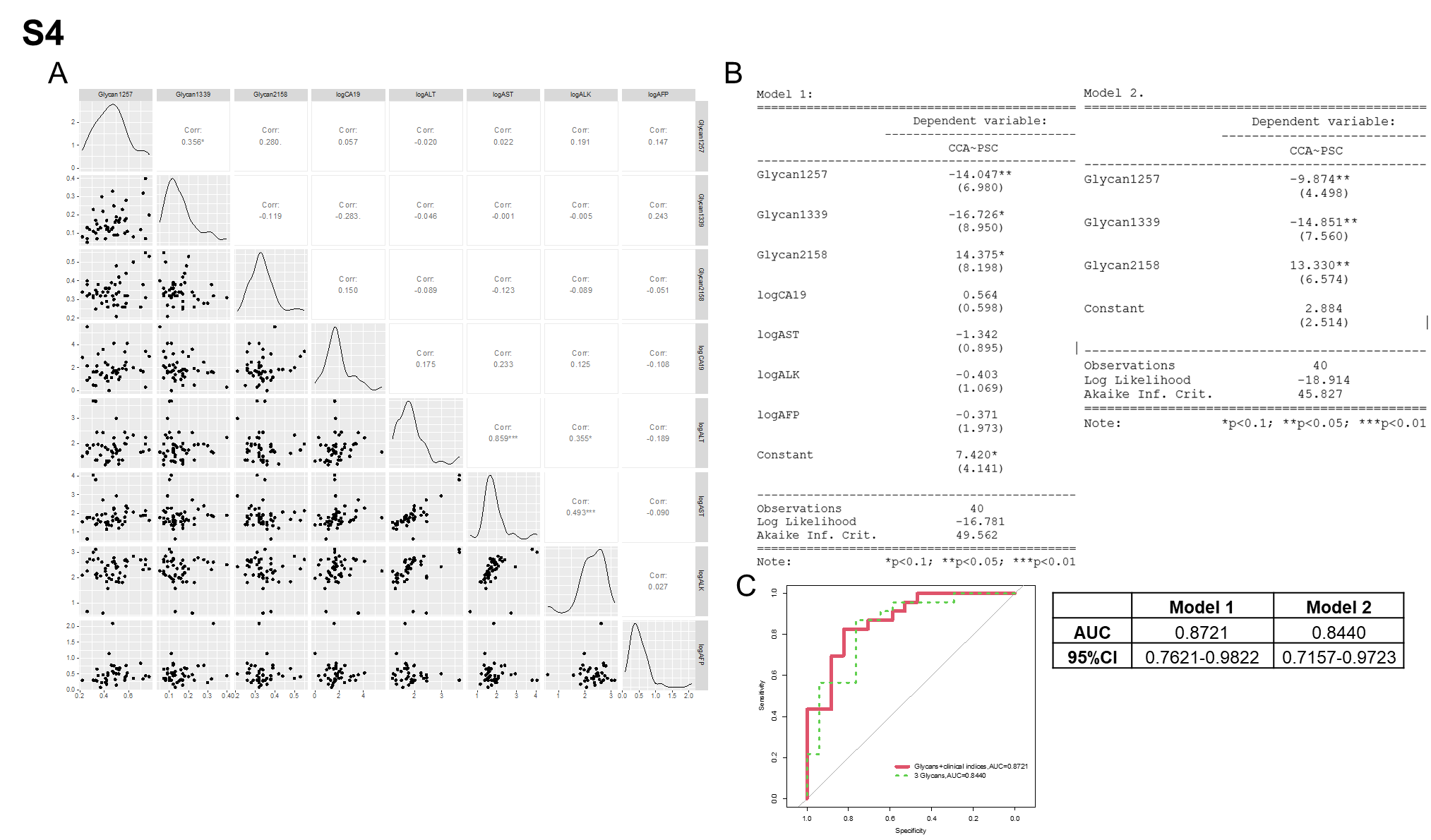


**Figure Supplemental 4**. **A.** Scatterplots and correlations between N-glycans of interest (1339, 1257, 2158) and clinical information available (ALT, AST, ALK, and AFP). **B.** Multivariate model-Multiple logistic regression in CCA and PSC serum samples (n=40). Model 1: three N-glycans of interest and clinical information available (left panel). Model 2: Only the three N-glycans of interest (right panel). **C.** ROC curve of the combination of glycans and clinical information (red-solid line) and only glycans (green-dashed line) (left), classification performance table of the two models in B. p=0.5731, Delong’s test between model 1 and 2. CA19-9, ALT, AST, ALK, and AFP values were log-transformed for plotting and modeling convenience. logALT was removed from the multiple logistic regression analysis due to a high value of Variance inflation factor (VIF).
