# Supplementary material for: Analysis of N-linked Glycan Alterations in Tissue and Serum Reveals Promising Biomarkers for Intrahepatic Cholangiocarcinoma
Source: Cancer Res Commun. 2023 Mar 6;3(3):383–94. doi: 10.1158/2767-9764.CRC-22-0422 (PMC9987250; doi:10.1158/2767-9764.CRC-22-0422)
Supplement: Supplementary Figure SF5 — Relative intensity quantification of all N-glycans identified in serum (left row) and tissue (right row) analysis. Red font labeling for N-glycans follows the same trend between serum and tissue. [file crc-22-0422-s05.docx]

**S5**

Glycan1136 Glycan1257

Glycan1282 Glycan1298

Glycan1339 Glycan1419

Glycan1444 Glycan1485

Glycan1501 Glycan1581

Glycan1606 Glycan1611

Glycan1622 Glycan1647

Glycan1663 Glycan1688

Glycan1704 Glycan1743

Glycan1809 Glycan1825

Glycan1850 Glycan1866

Glycan1905 Glycan1954

Glycan1976 Glycan2012

Glycan2028 Glycan2100

Glycan2122 Glycan2158

Glycan2163 Glycan2174

Glycan2303 Glycan2319

Glycan2320 Glycan2325

Glycan2341 Glycan2361

Glycan2377 Glycan2393

Glycan2465 Glycan2487

Glycan2539 Glycan2654

Glycan2685 Glycan2706

Glycan2852

**Supplemental Figure 5.** Relative intensity quantification of all N-glycans identified in serum (left row) and tissue (right row) analysis. Red font labeling for N-glycans follows the same trend between serum and tissue.
