# Supplementary material for: Analysis of N-linked Glycan Alterations in Tissue and Serum Reveals Promising Biomarkers for Intrahepatic Cholangiocarcinoma
Source: Cancer Res Commun. 2023 Mar 6;3(3):383–94. doi: 10.1158/2767-9764.CRC-22-0422 (PMC9987250; doi:10.1158/2767-9764.CRC-22-0422)
Supplement: Supplementary Table ST1 — Peak list of N-glycans detected in tissue (TMA) and serum samples. [file crc-22-0422-s06.docx]

**Supplementary Table 1**

| **Data set** | **Observed mass (m/z)** | **Theoretical mass (m/z)** | **Mass error (ppm)** | **Composition** | **Proposed N-glycan** |
| --- | --- | --- | --- | --- | --- |
| TMA | 933.308 | 933.317 | 9.64 | Hex3HexNAc2 + 1Na | 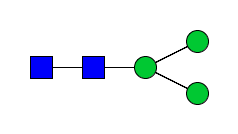 |
| Serum |  |  |  |  |  |
|  |  |  |  |  |  |
| TMA | 1079.365 | 1079.374 | 9.17 | Hex3dHex1HexNAc2 + 1Na | 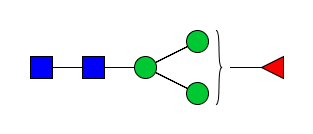 |
| Serum |  |  |  |  |  |
| TMA | 1095.363 | 1095.3698 | 6.21 | Hex4HexNAc2 + 1Na | 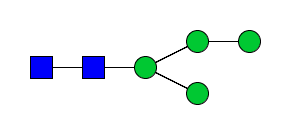 |
| Serum |  |  |  |  |  |
| TMA | 1136.381 | 1136.396 | 13.55 | Hex3HexNAc3 + 1Na | 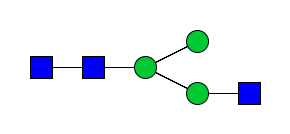 |
| Serum | 1136.394 |  | 1.76 |  |  |
| TMA | 1257.416 | 1257.4226 | 5.25 | Hex5HexNAc2 + 1Na | 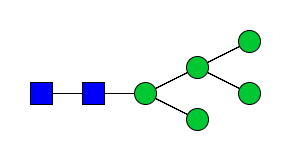 |
| Serum | 1257.424 |  | 0.80 |  |  |
| TMA | 1282.454 | 1282.4543 | 0.23 | Hex3dHex1HexNAc3 + 1Na | 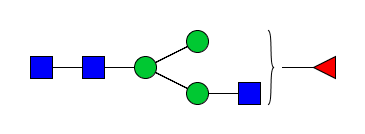 |
| Serum | 1282.453 |  | 0.78 |  |  |
| TMA | 1298.454 | 1298.449 | 3.67 | Hex4HexNAc3 + 1Na | 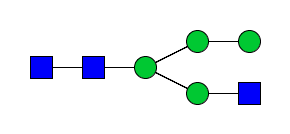 |
| Serum | 1298.448 |  | 0.77 |  |  |
| TMA | 1339.467 | 1339.476 | 6.57 | Hex3HexNAc4 + 1Na | 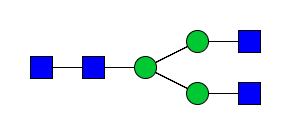 |
| Serum | 1339.478 |  | 1.49 |  |  |
| TMA | 1403.468 | 1403.480 | 8.91 | Hex5dHex1HexNAc2 + 1Na | 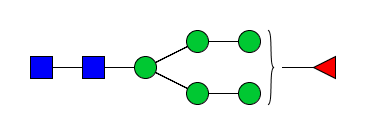 |
| Serum |  |  |  |  |  |
| TMA | 1419.478 | 1419.475 | 1.83 | Hex6HexNAc2 + 1Na | 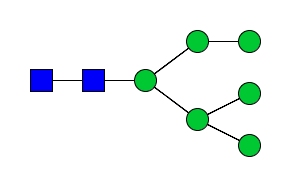 |
| Serum | 1419.472 |  | 2.82 |  |  |
| TMA | 1444.513 | 1444.507 | 4.09 | Hex4dHex1HexNAc3 + 1Na | 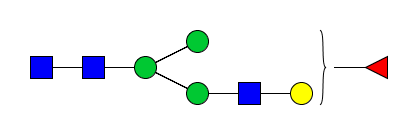 |
| Serum | 1444.509 |  | 1.38 |  |  |
| TMA | 1460.502 | 1460.502 | 8.90 | Hex5HexNAc3 + 1Na | 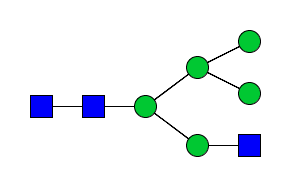 |
| Serum |  |  |  |  |  |
| TMA | 1485.527 | 1485.533 | 4.51 | Hex3dHex1HexNAc4 + 1Na | 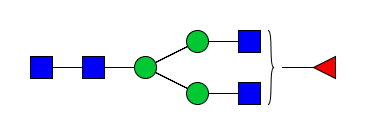 |
| Serum | 1485.535 |  | 0.67 |  |  |
| TMA | 1501.527 | 1501.528 | 1.07 | Hex4HexNAc4 + 1Na | 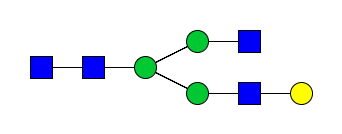 |
| Serum | 1501.530 |  | 0.67 |  |  |
| TMA | 1542.553 | 1542.510 | 1.36 | Hex3HexNAc5 + 1Na | 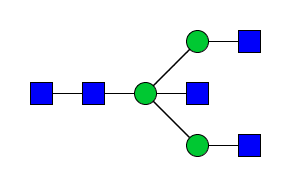 |
| Serum |  |  |  |  |  |
| TMA | 1581.526 | 1581.528 | 1.39 | Hex7HexNac2 + 1Na | 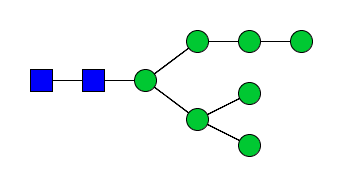 |
| Serum | 1581.525 |  | 1.90 |  |  |
| TMA | 1606.562 | 1606.559 | 1.30 | Hex5dHex1HexNac3 + 1Na | 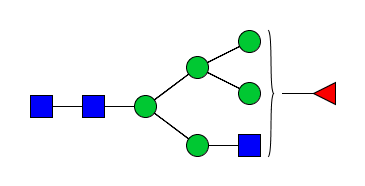 |
| Serum |  |  |  |  |  |
| TMA | 1611.531 | 1611.526 | 2.73 | Hex4HexNAc3NeuAc1+ 2Na | 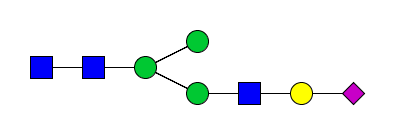 |
| Serum | 1611.523 |  | 2.48 |  |  |
| TMA | 1622.553 | 1622.554 | 1.11 | Hex6HexNac3 + 1Na | 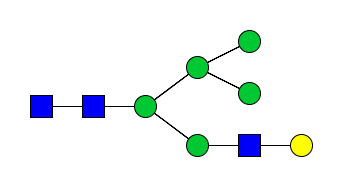 |
| Serum | 1622.551 |  | 2.47 |  |  |
| TMA | 1647.583 | 1647.586 | 2.13 | Hex4dHex1HexNAc4 + 1Na | 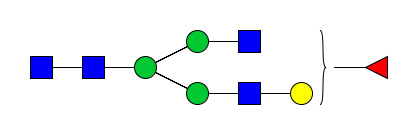 |
| Serum | 1647.584 |  | 1.82 |  |  |
| TMA | 1663.589 | 1663.581 | 4.57 | Hex5HexNAc4 + 1Na | 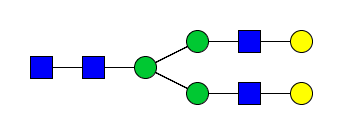 |
| Serum | 1663.585 |  | 2.40 |  |  |
| TMA | 1688.615 | 1688.613 | 1.19 | Hex3dHex1HexNAc5 + 1Na | 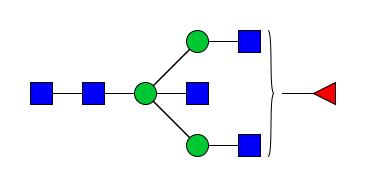 |
| Serum | 1688.617 |  | 2.37 |  |  |
| TMA | 1704.607 | 1704.607 | 0.53 | Hex4HexNAc5 + 1Na | 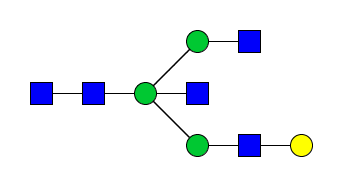 |
| Serum | 1704.607 |  | 0.59 |  |  |
| TMA | 1743.596 | 1743.581 | 8.60 | Hex8HexNAc2 + 1Na | 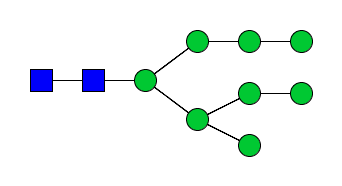 |
| Serum | 1743.579 |  | 1.15 |  |  |
| TMA |  | 1757.585 |  | Hex4dHex1HexNAc3NeuAc1 + 2Na | 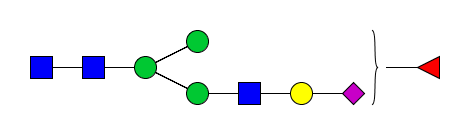 |
| Serum | 1757.577 |  | 4.55 |  |  |
| TMA | 1793.638 | 1793.644 | 3.57 | Hex4dHex2HexNAc4 + 1Na | 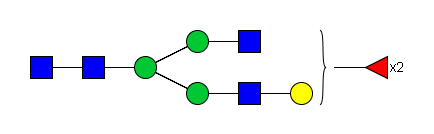 |
| Serum |  |  |  |  |  |
| TMA | 1809.629 | 1809.639 | 5.69 | Hex5dHex1HexNAc4 + 1Na | 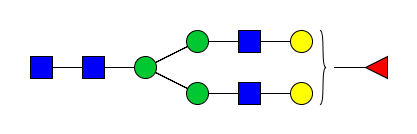 |
| Serum | 1809.643 |  | 2.21 |  |  |
| TMA | 1825.624 | 1825.634 | 5.59 | Hex6HexNAc4 + 1Na | 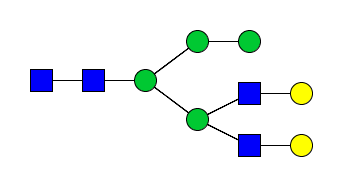 |
| Serum | 1825.638 |  | 2.19 |  |  |
| TMA |  | 1837.541 |  | Hex4dHex1HexNAc3NeuAc1 + 2Na2 + 1SO3 | 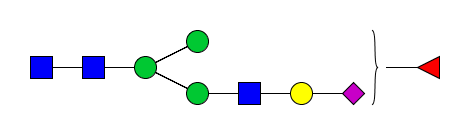 |
| Serum | 1837.571 |  | 16.33 |  |  |
| TMA | 1850.667 | 1850.665 | 0.59 | Hex4dHex1HexNAc5 + 1Na | 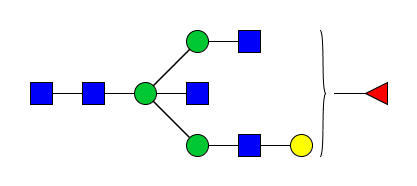 |
| Serum | 1850.667 |  | 0.54 |  |  |
| TMA | 1866.653 | 1866.661 | 4.18 | Hex5HexNAc5 + 1Na | 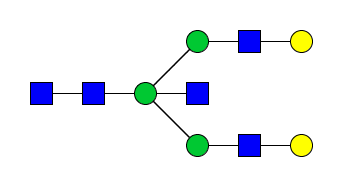 |
| Serum | 1866.667 |  | 3.21 |  |  |
| TMA |  | 1889.596 |  | Hex5dHex1HexNAc4 + 1Na + 1SO3 | 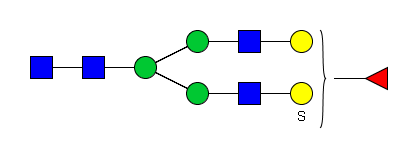 |
| Serum | 1889.627 |  | 16.41 |  |  |
| TMA | 1891.691 | 1891.692 | 0.74 | Hex3dHex1HexNAc6 + 1Na | 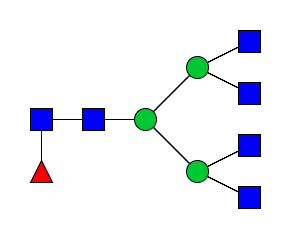 |
| Serum |  |  |  |  |  |
| TMA | 1905.644 | 1905.634 | 5.35 | Hex9HexNAc2 + 1Na | 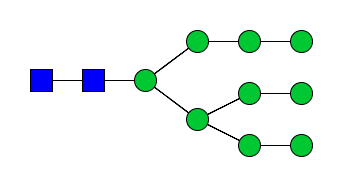 |
| Serum | 1905.634 |  | 0.00 |  |  |
| TMA |  | 1911.586 |  | Hex5dHex1HexNAc4 + 2Na2 + 1SO3 | 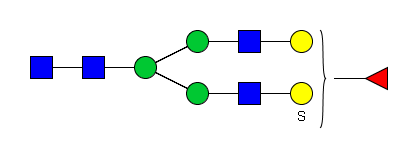 |
| Serum | 1911.579 |  | 0.52 |  |  |
| TMA |  | 1919.637 |  | Hex5dHex1HexNAc3NeuAc1 + 2Na | 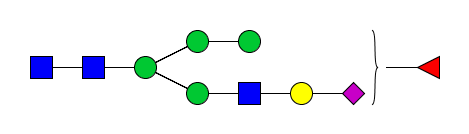 |
| Serum | 1919.653 |  | 8.33 |  |  |
| TMA | 1954.668 | 1954.677 | 4.50 | Hex5HexNAc4NeuAc1 + 1Na | 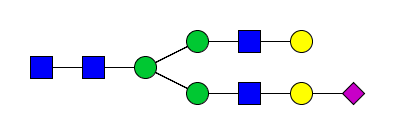 |
| Serum | 1954.676 |  | 0.51 |  |  |
| TMA | 1955.693 | 1955.697 | 2.15 | Hex5dHex2HexNAc4 + 1Na | 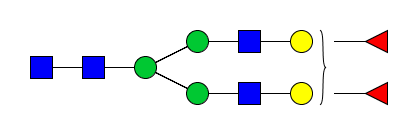 |
| Serum |  |  |  |  |  |
| TMA | 1971.686 | 1971.692 | 3.09 | Hex6dHex1HexNAc4 + 1Na | 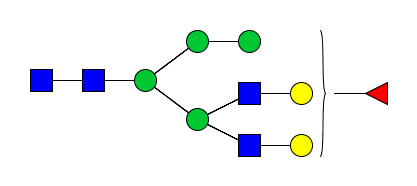 |
| Serum |  |  |  |  |  |
| TMA | 1976.658 | 1976.666 | 4.35 | Hex5HexNAc4NeuAc1 + 2Na | 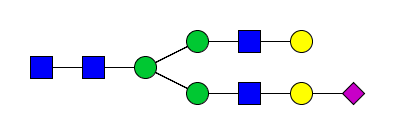 |
| Serum | 1976.662 |  | 1.52 |  |  |
| TMA | 1996.720 | 1996.723 | 1.90 | Hex4dHex2HexNAc5 + 1Na | 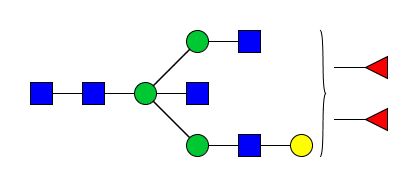 |
| Serum |  |  |  |  |  |
| TMA | 2012.732 | 2012.719 | 6.61 | Hex5dHex1HexNAc5 + 1Na | 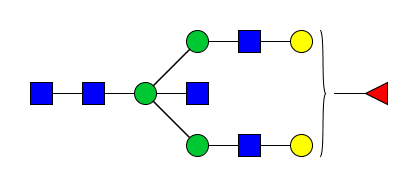 |
| Serum | 2012.715 |  | 1.99 |  |  |
| TMA | 2028.702 | 2028.714 | 5.72 | Hex6HexNAc5 + 1Na | 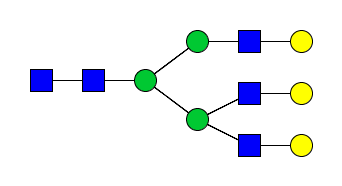 |
| Serum | 2028.719 |  | 2.46 |  |  |
| TMA | 2053.757 | 2053.745 | 5.74 | Hex4dHex1HexNAc6 + 1Na | 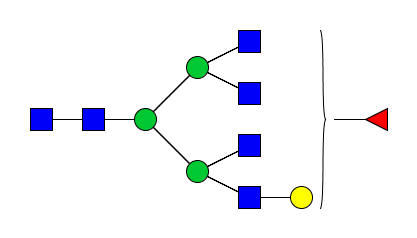 |
| Serum |  |  |  |  |  |
| TMA | 2100.754 | 2100.735 | 9.79 | Hex5dHex1HexNAc4NeuAc1 + 1Na | 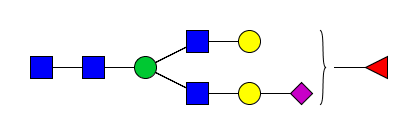 |
| Serum | 2100.740 |  | 2.38 |  |  |
| TMA | 2101.755 | 2101.7551 | 0.05 | Hex5dHex3HexNAc4 + 1Na | 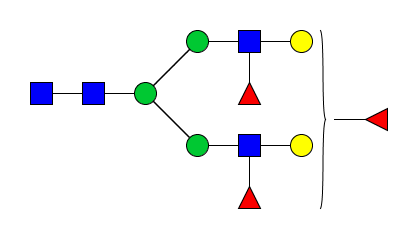 |
| Serum |  |  |  |  |  |
| TMA | 2122.714 | 2122.724 | 4.95 | Hex5dHex1HexNAc4NeuAc1 + 2Na | 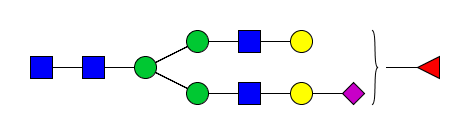 |
| Serum | 2122.723 |  | 2.83 |  |  |
| TMA | 2142.763 | 2142.781 | 8.73 | Hex4dHex3HexNAc5 + 1Na | 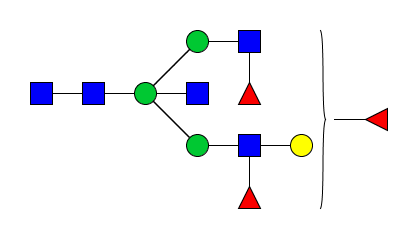 |
| Serum |  |  |  |  |  |
| TMA |  | 2157.756 |  | Hex5HexNAc5NeuAc1 + 1Na | 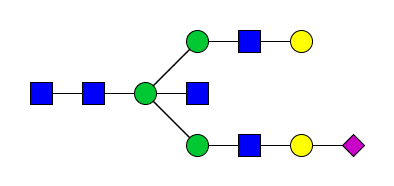 |
| Serum | 2157.779 |  | 10.66 |  |  |
| TMA | 2158.786 | 2158.776 | 4.35 | Hex5dHex2HexNAc5 + 1Na | 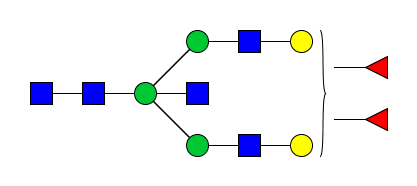 |
| Serum | 2158.783 |  | 2.78 |  |  |
| TMA | 2163.757 | 2163.743 | 6.38 | Hex4dHex1HexNAc5NeuAc1 + 2Na | 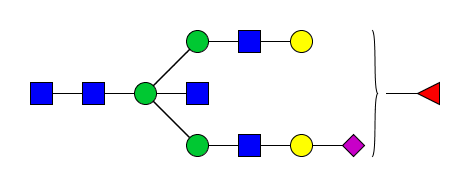 |
| Serum | 2163.743 |  | 0.00 |  |  |
| TMA | 2174.779 | 2174.771 | 3.45 | Hex6dHex1HexNAc5 + 1Na | 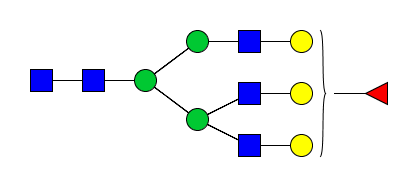 |
| Serum | 2174.774 |  | 0.45 |  |  |
| TMA | 2215.789 | 2215.798 | 4.06 | Hex5dHex1HexNAc6 + 1Na | 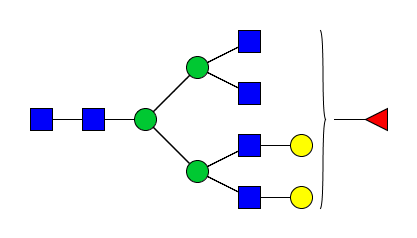 |
| Serum |  |  |  |  |  |
| TMA | 2231.797 | 2231.793 | 1.79 | Hex6HexNAc6 + 1Na | 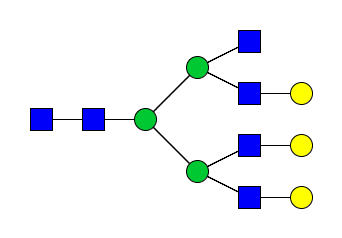 |
| Serum |  |  |  |  |  |
| TMA |  | 2245.772 |  | Hex5HexNAc4NeuAc2 + 1Na | 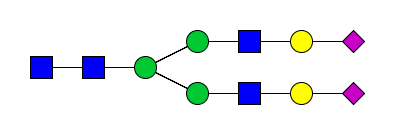 |
| Serum | 2245.773 |  | 0.45 |  |  |
| TMA | 2267.766 | 2267.762 | 1.76 | Hex5HexNAc4NeuAc2 + 2Na | 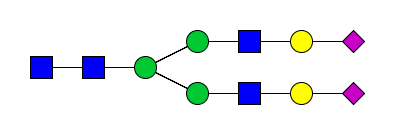 |
| Serum | 2267.753 |  | 0.44 |  |  |
| TMA | 2268.767 | 2268.782 | 6.79 | Hex5dHex2HexNAc4NeuAc1 + 2Na | 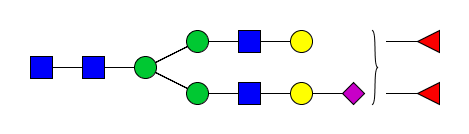 |
| Serum |  |  |  |  |  |
| TMA | 2289.721 | 2289.732 | 5.07 | Hex5HexNAc4NeuAc2 + 3Na | 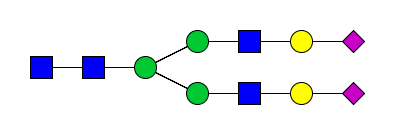 |
| Serum | 2289.743 |  | 3.06 |  |  |
| TMA | 2303.821 | 2303.814 | 2.99 | Hex5dHex1HexNAc5NeuAc1 + 1Na | 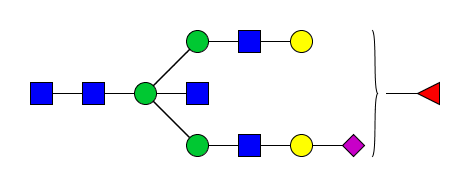 |
| Serum | 2303.822 |  | 3.47 |  |  |
| TMA | 2304.831 | 2304.835 | 1.52 | Hex5dHex3HexNAc5 + 1Na | 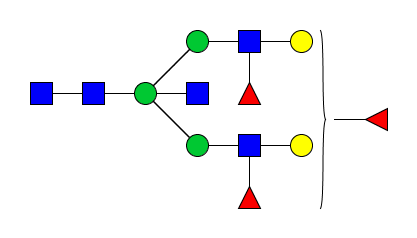 |
| Serum |  |  |  |  |  |
| TMA | 2319.809 | 2319.809 | 0.00 | Hex6HexNAc5NeuAc1 + 1Na | 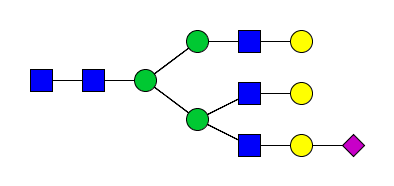 |
| Serum | 2319.808 |  | 0.43 |  |  |
| TMA | 2320.820 | 2320.829 | 4.05 | Hex6dHex2HexNAc5 + 1Na | 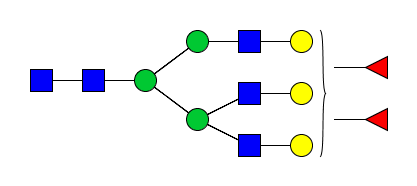 |
| Serum | 2320.829 |  | 0.00 |  |  |
| TMA | 2325.816 | 2325.803 | 5.20 | Hex5dHex1HexNAc5NeuAc1 + 2Na | 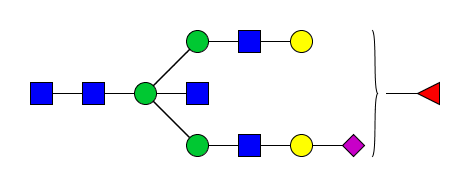 |
| Serum | 2325.801 |  | 2.15 |  |  |
| TMA | 2341.809 | 2341.798 | 8.16 | Hex6HexNAc5NeuAc1 + 2Na | 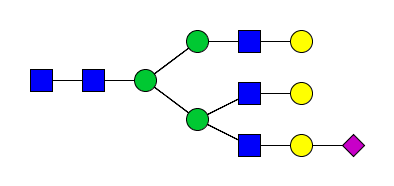 |
| Serum | 2341.799 |  | 3.49 |  |  |
| TMA | 2361.849 | 2361.855 | 2.96 | Hex5dHex2HexNAc6 + 1Na | 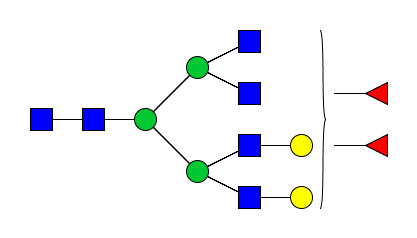 |
| Serum | 2361.823 |  | 13.97 |  |  |
| TMA | 2377.844 | 2377.850 | 2.90 | Hex6dHex1HexNAc6 + 1Na | 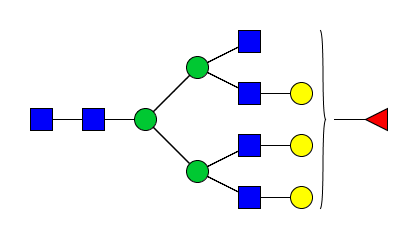 |
| Serum | 2377.859 |  | 3.36 |  |  |
| TMA | 2393.833 | 2393.845 | 5.35 | Hex7HexNAc6 + 1Na | 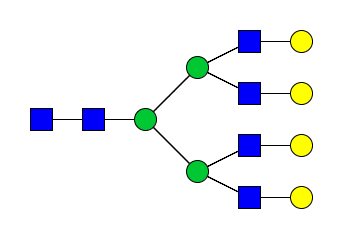 |
| Serum | 2393.842 |  | 1.67 |  |  |
| TMA | 2413.797 | 2413.819 | 9.49 | Hex5dHex1HexNAc4NeuAc2 + 2Na | 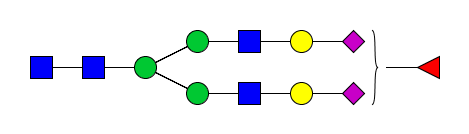 |
| Serum | 2413.818 |  | 2.49 |  |  |
| TMA | 2435.789 | 2435.809 | 8.49 | Hex5dHex1HexNAc4NeuAc2 + 3Na | 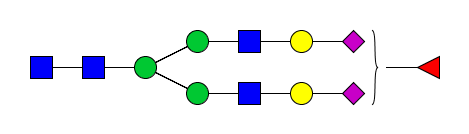 |
| Serum | 2435.798 |  | 1.64 |  |  |
| TMA | 2465.870 | 2465.867 | 1.26 | Hex6dHex1HexNAc5NeuAc1 + 1Na | 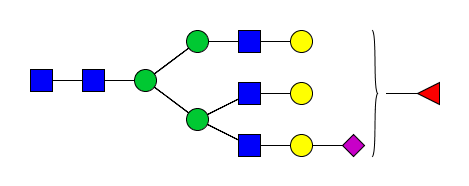 |
| Serum | 2465.870 |  | 1.22 |  |  |
| TMA | 2466.860 | 2466.887 | 11.07 | Hex6dHex3HexNAc5 + 1Na | 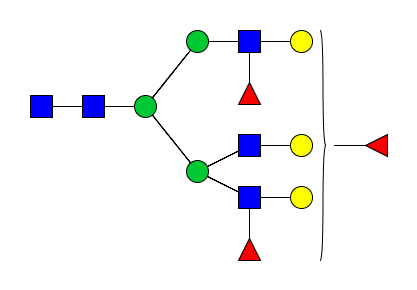 |
| Serum |  |  |  |  |  |
| TMA | 2487.860 | 2487.856 | 1.33 | Hex6dHex1HexNAc5NeuAc1 + 2Na | 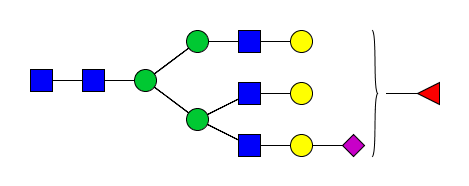 |
| Serum | 2487.852 |  | 1.21 |  |  |
| TMA | 2507.900 | 2507.913 | 5.54 | Hex5dHex3HexNAc6 + 1Na | 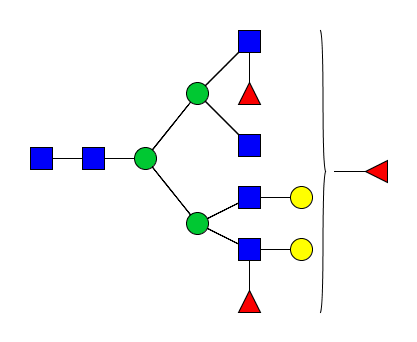 |
| Serum |  |  |  |  |  |
| TMA | 2523.918 | 2523.908 | 3.65 | Hex6dHex2HexNAc6 + 1Na | 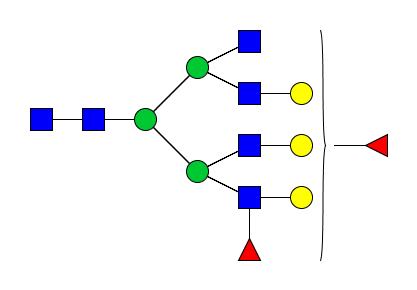 |
| Serum |  |  |  |  |  |
| TMA | 2539.895 | 2539.903 | 3.43 | Hex7dHex1HexNAc6 + 1Na | 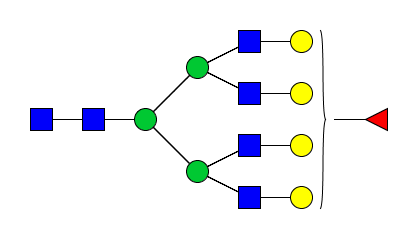 |
| Serum | 2539.900 |  | 1.57 |  |  |
| TMA |  | 2550.790 |  | Hex5HexNAc5NeuAc2 + 2Na + 1SO3 | 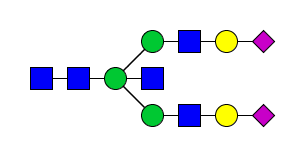 |
| Serum | 2550.717 |  | 28.78 |  |  |
| TMA |  | 2572.772 |  | Hex5HexNAc5NeuAc2 + 3Na + 1SO3 | 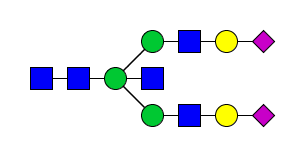 |
| Serum | 2572.696 |  | 29.54 |  |  |
| TMA | 2580.930 | 2580.929 | 0.45 | Hex6dHex1HexNAc7 + 1Na | 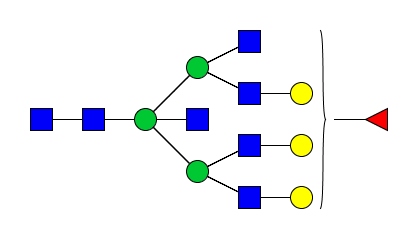 |
| Serum |  |  |  |  |  |
| TMA | 2596.935 | 2596.925 | 3.77 | Hex7HexNAc7 + 1Na | 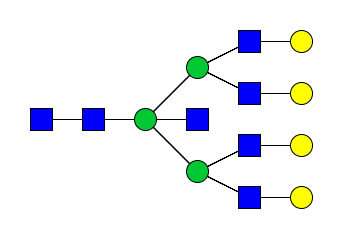 |
| Serum |  |  |  |  |  |
| TMA |  | 2610.904 |  | Hex6HexNAc5NeuAc2 + 1Na | 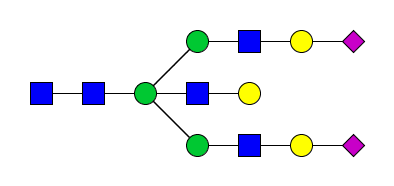 |
| Serum | 2610.912 |  | 3.06 |  |  |
| TMA | 2612.925 | 2612.945 | 7.73 | Hex6dHex4HexNAc5 + 1Na | 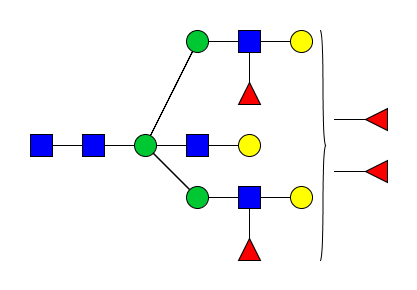 |
| Serum |  |  |  |  |  |
| TMA |  | 2616.892 |  | Hex5dHex1HexNAc5NeuAc2 + 2Na | 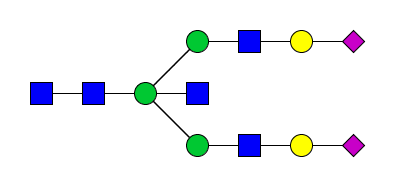 |
| Serum | 2616.898 |  | 3.06 |  |  |
| TMA | 2632.892 | 2632.894 | 8.58 | Hex6HexNAc5NeuAc2 + 2Na | 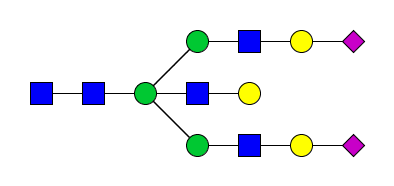 |
| Serum | 2632.896 |  | 3.80 |  |  |
| TMA | 2633.913 | 2633.914 | 0.61 | Hex6dHex2HexNAc5NeuAc1 + 2Na | 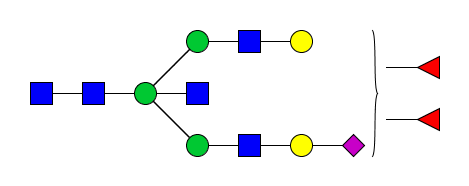 |
| Serum |  |  |  |  |  |
| TMA |  | 2638.889 |  | Hex5dHex1HexNAc5NeuAc2 + 3Na | 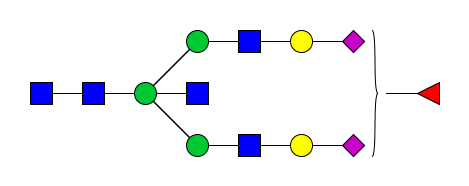 |
| Serum | 2638.880 |  | 2.65 |  |  |
| TMA |  | 2654.868 |  | Hex6HexNAc5NeuAc2 + 3Na | 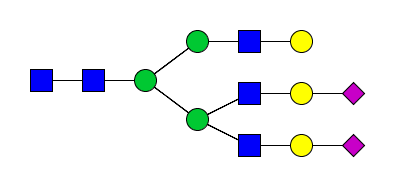 |
| Serum | 2654.867 |  | 0.38 |  |  |
| TMA | 2669.950 | 2669.966 | 6.25 | Hex6dHex3HexNAc6 + 1Na | 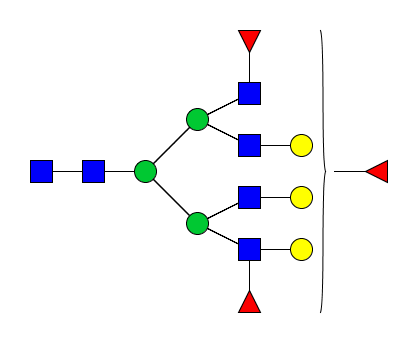 |
| Serum |  |  |  |  |  |
| TMA | 2684.937 | 2684.941 | 1.56 | Hex7HexNAc6NeuAc1 + 1Na | 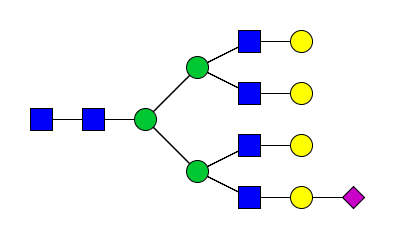 |
| Serum |  |  |  |  |  |
| TMA | 2685.952 | 2685.961 | 3.57 | Hex7dHex2HexNAc6 + 1Na | 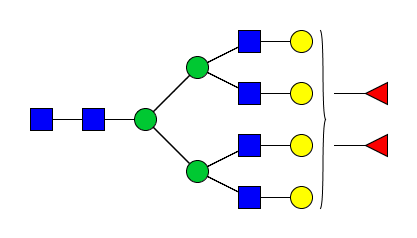 |
| Serum | 2685.964 |  | 0.89 |  |  |
| TMA | 2706.945 | 2706.931 | 5.17 | Hex7HexNAc6NeuAc1 + 2Na | 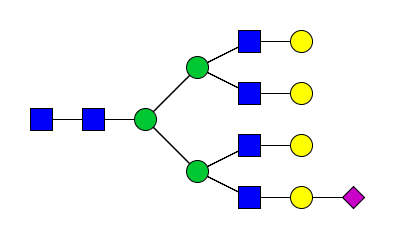 |
| Serum | 2706.928 |  | 1.85 |  |  |
| TMA |  | 2756.962 |  | Hex6dHex1HexNAc5NeuAc2 + 2Na | 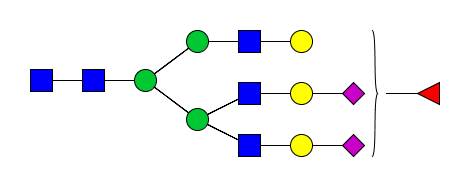 |
| Serum | 2756.962 |  | 0.00 |  |  |
| TMA | 2758.967 | 2758.978 | 3.99 | Hex8HexNAc7 + 1Na | 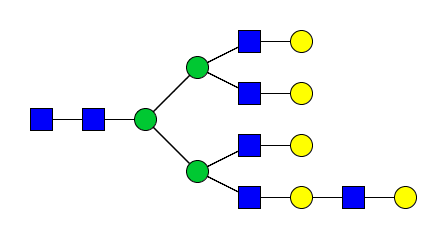 |
| Serum |  |  |  |  |  |
| TMA |  | 2800.926 |  | Hex6dHex1HexNAc5NeuAc2 + 3Na | 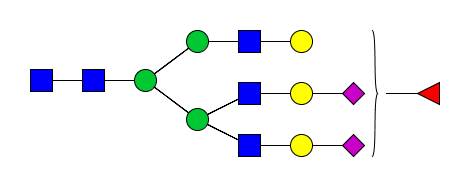 |
| Serum | 2800.924 |  | 0.71 |  |  |
| TMA | 2815.015 | 2815.004 | 3.84 | Hex6dHex2HexNAc6NeuAc1 + 1Na | 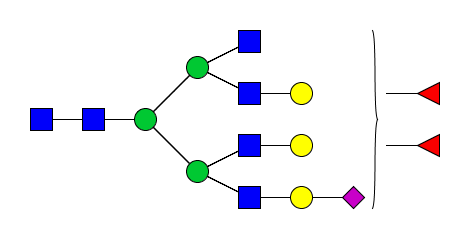 |
| Serum |  |  |  |  |  |
| TMA | 2830.994 | 2830.999 | 1.80 | Hex7dHex1HexNAc6NeuAc1 + 1Na | 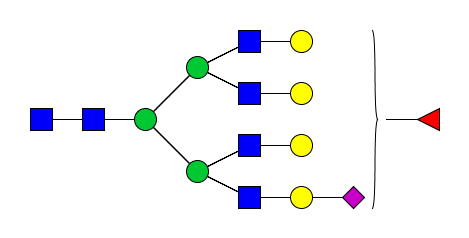 |
| Serum |  |  |  |  |  |
| TMA | 2835.960 | 2835.974 | 4.79 | Hex7dHex1HexNAc6NeuAc1 + 2Na | 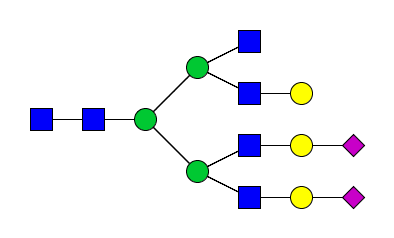 |
| Serum |  |  |  |  |  |
| TMA | 2836.995 | 2836.994 | 0.35 | Hex6dHex2HexNAc6NeuAc1 + 2Na | 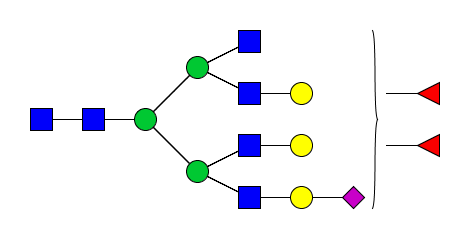 |
| Serum |  |  |  |  |  |
| TMA | 2852.998 | 2852.989 | 3.19 | Hex7dHex1HexNAc6NeuAc1 + 2Na | 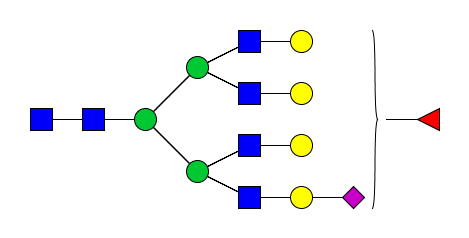 |
| Serum | 2853.024 |  | 15.07 |  |  |
| TMA | 2873.044 | 2873.046 | 0.73 | Hex6dHex3HexNAc7 + 1Na | 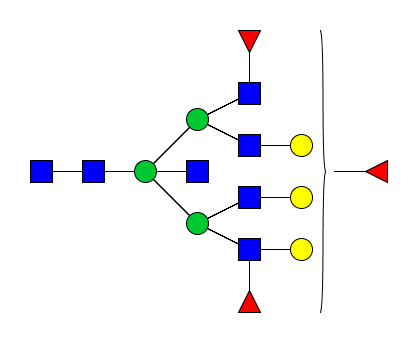 |
| Serum |  |  |  |  |  |
| TMA | 2889.028 | 2889.041 | 5.19 | Hex7dHex2HexNAc7 + 1Na | 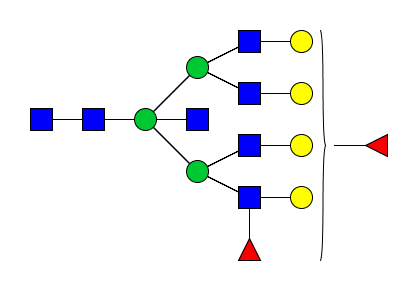 |
| Serum |  |  |  |  |  |
| TMA | 2905.034 | 2905.035 | 0.65 | Hex8dHex1HexNAc7 + 1Na |  |
| Serum |  |  |  |  |  |
| TMA |  | 2945.964 |  | Hex6HexNAc5NeuAc3 + 3Na |  |
| Serum | 2945.974 |  | 3.39 |  |  |
| TMA |  | 2967.946 |  | Hex6HexNAc5NeuAc3 + 4Na |  |
| Serum | 2967.959 |  | 4.45 |  |  |
| TMA | 2978.054 | 2978.077 | 7.86 | Hex7dHex4HexNAc6 + 1Na |  |
| Serum |  |  |  |  |  |
| TMA | 3035.102 | 3035.098 | 1.02 | Hex7dHex3HexNAc7 + 1Na |  |
| Serum |  |  |  |  |  |
| TMA |  | 3092.022 |  | Hex7dHex2HexNAc8 + 1Na |  |
| Serum | 3092.035 |  | 4.20 |  |  |
| TMA | 3108.130 | 3108.115 | 4.73 | Hex8dHex1HexNac8 + 1Na |  |
| Serum |  |  |  |  |  |
| TMA |  | 3114.004 |  | Hex6dHex1HexNAc5NeuAc3 + 4Na |  |
| Serum | 3114.012 |  | 2.57 |  |  |
| TMA | 3124.100 | 3124.110 | 3.26 | Hex9HexNAc8 + 1Na |  |
| Serum |  |  |  |  |  |
| TMA | 3197.141 | 3197.152 | 3.35 | Hex8dHex3HexNAc7 + 1Na |  |
| Serum |  |  |  |  |  |
| TMA | 3254.197 | 3254.173 | 7.31 | Hex8dHex2HexNAc8 + 1Na |  |
| Serum |  |  |  |  |  |
| TMA | 3270.171 | 3270.168 | 0.89 | Hex9dHex1HexNAc8 + 1Na |  |
| Serum |  |  |  |  |  |
| TMA | 3327.244 | 3327.217 | 7.99 | Hex7dHex5HexNAc7 + 1Na |  |
| Serum |  |  |  |  |  |
| TMA | 3343.220 | 3343.209 | 3.11 | Hex8dHex4HexNAc7 + 1Na |  |
| Serum |  |  |  |  |  |

**Supplementary Table 1**. Peak list of N-glycans detected in tissue (TMA) and serum samples.
