# Supplementary material for: Analysis of N-linked Glycan Alterations in Tissue and Serum Reveals Promising Biomarkers for Intrahepatic Cholangiocarcinoma
Source: Cancer Res Commun. 2023 Mar 6;3(3):383–94. doi: 10.1158/2767-9764.CRC-22-0422 (PMC9987250; doi:10.1158/2767-9764.CRC-22-0422)
Supplement: Supplementary Table ST2 — Lists individual N-glycans, ALT (Alanine transaminase), AST (aspartate aminotransferase), ALK (Alkaline phosphatase), and AFP (Alpha-fetoprotein) of analysis to differentiate patients with iCCA from PSC. 1Possible biomarkers 2n: number of total patients 3distribution of iCCA (n=30) and PSC (n=17) patients. 4AUC (Area Under the Curve) 5CI (confidence interval) 6p value. Some iCCA patients were excluded from the analysis due to missing clinical information. [file crc-22-0422-s07.docx]

**Supplementary Table 2**


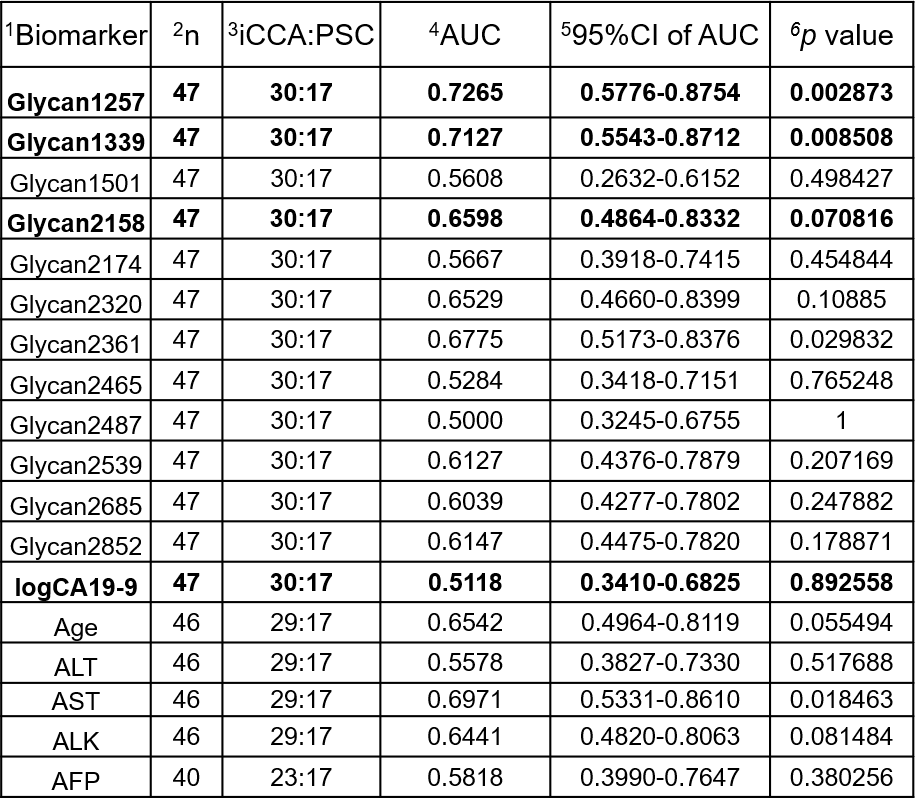


**Supplementary Table 2**. Lists individual N-glycans, ALT (Alanine transaminase), AST (aspartate aminotransferase), ALK (Alkaline phosphatase), and AFP (Alpha-fetoprotein) of analysis to differentiate patients with iCCA from PSC.

^1^Possible biomarkers ^2^n: number of total patients ^3^distribution of iCCA (n=30) and PSC (n=17) patients. ^4^AUC (Area Under the Curve) ^5^CI (confidence interval) ^6^p value. Some iCCA patients were excluded from the analysis due to missing clinical information.
